# A novel nutritional index and risk of edentulism: evidence from cross-sectional, prospective, and trajectory analyses

**DOI:** 10.1186/s12944-026-02860-2

**Published:** 2026-01-14

**Authors:** Qi Luo, Qian Yang, Yue Cao

**Affiliations:** 1 Department of Periodontology, Hospital of Stomatology, Zhongshan City, No. 73 Hubin Road, Shiqi Street, Zhongshan City, Guangdong Province 528400 China; 2 The Second School of Clinical Medicine, Southern Medical University, Guangzhou, Guangdong 510515 China; 3 Department of Cardiology, The Affiliated Hospital, Southwest Medical University, Luzhou, Sichuan China

**Keywords:** Tooth loss, Triglyceride–Cholesterol–Body weight index, Nutritional status, Prospective studies, Longitudinal studies

## Abstract

**Background:**

Nutritional status, recognized as a modifiable determinant of oral health, has recently gained increasing attention in the context of edentulism. The triglyceride–total cholesterol–body weight index (TCBI) is a novel nutritional indicator derived from routine clinical measures. However, its association with edentulism remains unclear. This study was designed to assess the association between TCBI and edentulism risk.

**Methods:**

This study utilized survey data provided by the China Health and Retirement Longitudinal Study (CHARLS). Three analyses were performed: cross-sectional (*n* = 9,686), prospective (participants without baseline edentulism, *n* = 8,568), and trajectory analyses (TCBI trajectories and incident edentulism, *n* = 4,921). Logistic regression, Cox proportional hazards models, group-based trajectory modeling, and restricted cubic spline analyses were applied. Sensitivity analyses using cumulative TCBI during follow-up were also conducted.

**Results:**

In the cross-sectional analysis, individuals in the highest TCBI tertile demonstrated a significantly lower risk of prevalent edentulism (adjusted OR = 0.80, 95% CI: 0.65–0.97). In the prospective analysis, higher TCBI levels were independently associated with a reduced risk of incident edentulism (adjusted HR = 0.85, 95% CI: 0.77–0.92). Trajectory modeling demonstrated that individuals with persistently high TCBI had the lowest risk of incident edentulism (adjusted HR = 0.59, 95% CI: 0.40–0.89). These associations remained robust in sensitivity analyses.

**Conclusion:**

TCBI was consistently and inversely associated with edentulism across cross-sectional, prospective, and trajectory analyses. As a readily obtainable nutritional index, TCBI may have clinical utility for the early identification and risk prediction of edentulism.

**Supplementary Information:**

The online version contains supplementary material available at 10.1186/s12944-026-02860-2.

## Introduction

Edentulism, defined as the complete loss of all natural teeth, has been recognized as the ultimate marker of oral health burden [1]. According to the World Health Organization, approximately 23% of individuals aged 60 years and above are completely edentulous. Edentulism not only compromises masticatory function and nutritional intake but also contributes to speech difficulties, social withdrawal, and psychological decline, imposing a substantial burden on older adults [2, 3]. Recent studies have indicated that tooth loss represents not merely a localized oral condition but also a significant indicator of systemic health risks. Evidence has shown strong associations between tooth loss and cardiovascular disease, frailty, cognitive decline, and increased all-cause mortality [4–6]. These findings highlight that the prevention and management of tooth loss extend beyond dental care, becoming a crucial issue in public health and systemic disease prevention. Identifying modifiable risk factors for edentulism and promoting early screening and prevention are therefore essential for improving population health.

The development of edentulism is typically a cumulative process influenced by multiple factors, including dental caries, periodontal disease, poor oral hygiene, smoking, low socioeconomic status, and limited access to dental care [7, 8]. Beyond these well-established determinants, nutritional status—a modifiable lifestyle factor—has recently been recognized as a potential contributor to tooth preservation and oral tissue homeostasis [9]. Adequate nutrition supports collagen synthesis, bone remodeling, and immune defense, maintaining the stability of periodontal and alveolar structures. Conversely, malnutrition is associated with impaired collagen formation, reduced immune function, and accelerated alveolar bone resorption, which may hasten the transition from partial tooth loss to complete edentulism [10–12]. Epidemiological studies have further shown that individuals with poorer baseline nutritional status are more likely to develop extensive tooth loss or edentulism, suggesting that declining nutrition may play a critical role in this progressive process [13, 14]. Thus, edentulism not only represents the terminal stage of dental loss but may also reflect the cumulative effects of long-term nutritional deficiency.

However, traditional single-nutrient or anthropometric indicators are insufficient to comprehensively assess an individual’s overall nutritional reserves [15, 16]. The triglyceride–cholesterol–body weight index (TCBI), a composite nutritional indicator derived from routine clinical parameters, provides an objective reflection of energy and nutritional status [17]. Previous research has demonstrated that low TCBI levels are closely associated with cardiovascular events, adverse cancer outcomes, and higher all-cause mortality, underscoring its value as an integrated marker of systemic nutrition [18–20]. Nonetheless, no studies to date have investigated the relationship between TCBI and edentulism, and its potential implications for oral health remain unexplored.

Building on this background, the present study hypothesized that lower TCBI levels might be associated with an increased risk of edentulism. Using nationally representative data from the China Health and Retirement Longitudinal Study (CHARLS) [21], a multi-layered analytical framework was applied to evaluate the association between TCBI and edentulism. Cross-sectional analysis was conducted to examine the relationship between baseline TCBI and prevalent edentulism, whereas prospective cohort analysis was applied to assess the predictive value of baseline TCBI for incident edentulism during follow-up. In addition, trajectory analysis based on repeated TCBI measurements was performed to characterize distinct longitudinal patterns of nutritional status and to evaluate their associations with subsequent edentulism risk. Meanwhile, cumulative TCBI derived from the same repeated measurements was incorporated as a sensitivity analysis to further assess the robustness of the longitudinal findings under alternative exposure definitions. By integrating these complementary analytical designs, the present study sought to clarify the population-level association between overall nutritional status and the development of edentulism, provide new epidemiological evidence for the role of nutrition in maintaining oral health, and inform early screening and individualized prevention strategies.

## Methods

### Data source

Information for this study was sourced from CHARLS, a longitudinal cohort that collects data from Chinese adults aged 45 years and older and is designed to achieve national population coverage [21]. The initial round of data collection took place in 2011–2012 and was based on a population-based sampling framework. Subsequent follow-up waves were carried out every two to three years to gather updated information on participants’ demographic characteristics, socioeconomic conditions, health status, and lifestyle behaviors.

### Study design and population

**Fig. 1:**
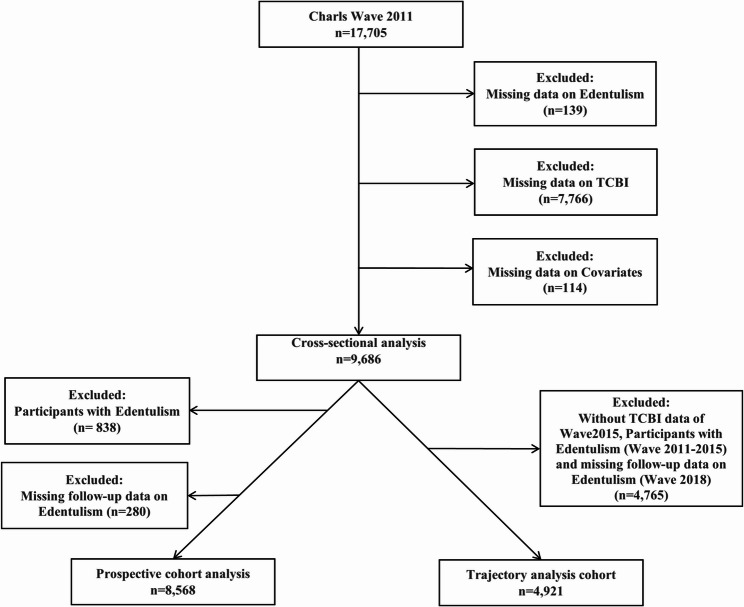
Flow chart for inclusion and exclusion of the study population

At baseline (wave 2011), 17,705 participants were enrolled. For the cross-sectional analysis, participants lacking data on edentulism (*n* = 139), TCBI (*n* = 7,766), or key covariates (*n* = 114) were excluded, leaving 9,686 participants. The prospective and trajectory analyses were subsequently conducted within this remaining population. For the prospective analysis, participants with edentulism at baseline (*n* = 838) or without follow-up information (*n* = 280) were further excluded, yielding 8,568 participants. For the trajectory analysis, participants missing TCBI data in wave 2015, those who developed edentulism between wave 2011 and 2015, or those lacking wave 2018 follow-up data (*n* = 4,765) were excluded, resulting in 4,921 participants. A schematic overview of participant inclusion and exclusion is provided in Fig. 1.

### Exposure variable: TCBI

TCBI was derived according to the following equation [22]:

$$\:\mathrm{T}\mathrm{C}\mathrm{B}\mathrm{I}\:=\frac{\left(\mathrm{t}\mathrm{r}\mathrm{i}\mathrm{g}\mathrm{l}\mathrm{y}\mathrm{c}\mathrm{e}\mathrm{r}\mathrm{i}\mathrm{d}\mathrm{e}\mathrm{s}\:\left(\mathrm{m}\mathrm{g}/\mathrm{d}\mathrm{L}\right)\times\:\:\mathrm{t}\mathrm{o}\mathrm{t}\mathrm{a}\mathrm{l}\:\mathrm{c}\mathrm{h}\mathrm{o}\mathrm{l}\mathrm{e}\mathrm{s}\mathrm{t}\mathrm{e}\mathrm{r}\mathrm{o}\mathrm{l}\:\left(\mathrm{m}\mathrm{g}/\mathrm{d}\mathrm{L}\right)\times\:\:\mathrm{b}\mathrm{o}\mathrm{d}\mathrm{y}\:\mathrm{w}\mathrm{e}\mathrm{i}\mathrm{g}\mathrm{h}\mathrm{t}\:\left(\mathrm{k}\mathrm{g}\right)\right)}{1000}$$

### Outcome variable: edentulism

Edentulism was assessed through the oral health questionnaire using the item, “Have you lost all of your natural teeth?” A response of “yes” to this question indicated edentulism, whereas a response of “no” indicated non-edentulism [23]. Self-reported edentulism has been validated as a reliable measure in older populations and is widely adopted in large-scale epidemiological studies [24, 25].

### Covariates

Covariate selection was grounded in prior empirical evidence [21, 26] and informed by a directed acyclic graph (DAG) constructed to illustrate and evaluate the hypothesized causal relationships among TCBI, edentulism, and potential confounders (Supplementary Fig. 1). The final model included age, sex, residence (rural/urban), education (primary school, middle school, college or above, or no formal education), marital status (unmarried, married, or others), smoking status (yes/no), drinking status (yes/no), hypertension (ascertained through respondents’ prior medical diagnosis or elevated blood-pressure readings at the survey examination), and diabetes (identified on the basis of previously diagnosed disease or abnormal glucose measurements obtained during the survey). Multicollinearity was assessed using the generalized variance inflation factor (GVIF) (Supplementary Tables 1–2). To allow comparisons across predictors with differing degrees of freedom, GVIF^(1/(2·Df)) values were examined, and all covariates showed values below 2, indicating no evidence of meaningful multicollinearity [27].

### Statistical analysis

Baseline characteristics were compared according to edentulism status. Categorical variables were assessed using chi-square–based procedures. Continuous variables were analyzed according to their underlying data distributions. For group comparisons, Student’s t test or the Wilcoxon rank-sum test was applied to evaluate differences between two independent groups, whereas one-way analysis of variance or the Kruskal–Wallis test was employed for comparisons involving three or more groups, as dictated by distributional assumptions.

For the cross-sectional analysis, multivariable logistic regression was applied to evaluate the association between TCBI and edentulism. Restricted cubic spline (RCS) models were used to explore possible nonlinear associations between baseline TCBI and edentulism.

For the prospective cohort analysis, baseline TCBI (wave 2011) was used to evaluate its longitudinal association with incident edentulism during follow-up. Baseline TCBI (wave 2011) was categorized into tertiles (T1–T3) to ensure balanced subgroup sizes and facilitate comparison across nutritional strata. Multivariable Cox proportional hazards models quantified the hazard ratios (HRs) and corresponding 95% confidence intervals (CIs). RCS models were also applied to evaluate dose–response relationships in the longitudinal context.

For the trajectory analysis, repeated TCBI measurements from waves 2011 and 2015 were incorporated into group-based trajectory modeling (GBTM) to characterize long-term intraindividual changes and their associations with edentulism risk. Models with one to four trajectories were fitted, and a three-group solution (Low, Moderate, High) was selected after considering the lowest Bayesian Information Criterion (BIC), acceptable Average Posterior Probability of Assignment (APPA ≥ 0.70), minimum group size > 5%, and clinical interpretability [28, 29] (Supplementary Tables 3 and Supplementary Fig. 2). Multivariable Cox proportional hazards models were applied to determine whether higher TCBI levels corresponded to a reduced hazard of developing edentulism over time.

To verify the stability of the longitudinal results, a sensitivity analysis was carried out using cumulative TCBI as an alternative exposure metric. This analysis was performed within the same dataset used for the trajectory analysis, which includes two TCBI measurements (2011 and 2015) and complete follow-up information for incident edentulism. Cumulative TCBI was calculated by the two measurements, and Cox proportional hazards models were applied to re-evaluate the association with incident edentulism.

Statistical analyses adopted a two-sided testing strategy, with a *P* value threshold of 0.05 defining statistical significance. Analyses were carried out in R (version 4.4.0), with the relevant packages summarized in Supplementary Table 4.

## Results

### Cross-sectional analysis: participant characteristics

In the cross-sectional analysis, 9,686 individuals were eligible for inclusion, of whom 8,848 did not have edentulism and 838 were identified as having edentulism (Table 1). Those classified with edentulism showed markedly reduced TCBI values compared with individuals without edentulism (6.91 vs. 7.06, *P* < 0.001). The median age was substantially higher among participants with edentulism (69.00 vs. 58.00 years, *P* < 0.001). A greater proportion of individuals with edentulism resided in rural areas compared with those without edentulism (71.36% vs. 64.22%, *P* < 0.001). Regarding education, a markedly greater proportion of individuals with edentulism had no formal education (65.87% vs. 45.91%, *P* < 0.001). The proportion of married individuals was significantly lower among those with edentulism (74.46% vs. 89.46%, *P* < 0.001). For lifestyle factors, smoking prevalence was similar between groups (41.29% vs. 38.44%, *P* = 0.11), and alcohol consumption was less frequent in participants with edentulism (28.16% vs. 32.78%, *P* = 0.007). As for comorbidities, hypertension was more prevalent among those with edentulism (47.26% vs. 40.14%, *P* < 0.001), while diabetes was less common (3.70% vs. 5.96%, *P* = 0.009). No meaningful difference in sex distribution was detected between the two groups (*P* = 0.42).

**Table 1:** Baseline characteristics in the cross-sectional study

Characteristic	Edentulism		*P* value
	No (*n* = 8,848)	Yes (*n* = 838)	
^**#**^ **TCBI**	7.06 [6.61;7.56]	6.91 [6.47;7.36]	< 0.001
**Age**	58.00 [51.00;64.00]	69.00 [62.00;75.00]	< 0.001
**Sex**			0.42
Female	4785 (54.08%)	466 (55.61%)	
Male	4063 (45.92%)	372 (44.39%)	
**Education**			< 0.001
College or above	122 (1.38%)	6 (0.72%)	
Middle school	2627 (29.69%)	104 (12.41%)	
No formal education	4062 (45.91%)	552 (65.87%)	
Primary school	2037 (23.02%)	176 (21.00%)	
**Residence**			< 0.001
Rural	5682 (64.22%)	598 (71.36%)	
Urban	3166 (35.78%)	240 (28.64%)	
**Marital status**			< 0.001
Married	7915 (89.46%)	624 (74.46%)	
Others	879 (9.93%)	206 (24.58%)	
Unmarried	54 (0.61%)	8 (0.95%)	
**Smoke**			0.11
No	5447 (61.56%)	492 (58.71%)	
Yes	3401 (38.44%)	346 (41.29%)	
**Drinking**			0.007
No	5948 (67.22%)	602 (71.84%)	
Yes	2900 (32.78%)	236 (28.16%)	
**Hypertension**			< 0.001
No	5296 (59.86%)	442 (52.74%)	
Yes	3552 (40.14%)	396 (47.26%)	
**Diabetes**			0.009
No	8321 (94.04%)	807 (96.30%)	
Yes	527 (5.96%)	31 (3.70%)	

*TCBI* Triglyceride–Cholesterol–Body Weight Index

^#^ TCBI values were log-transformed

### Cross-sectional analysis: association between TCBI and edentulism

**Fig. 2:**
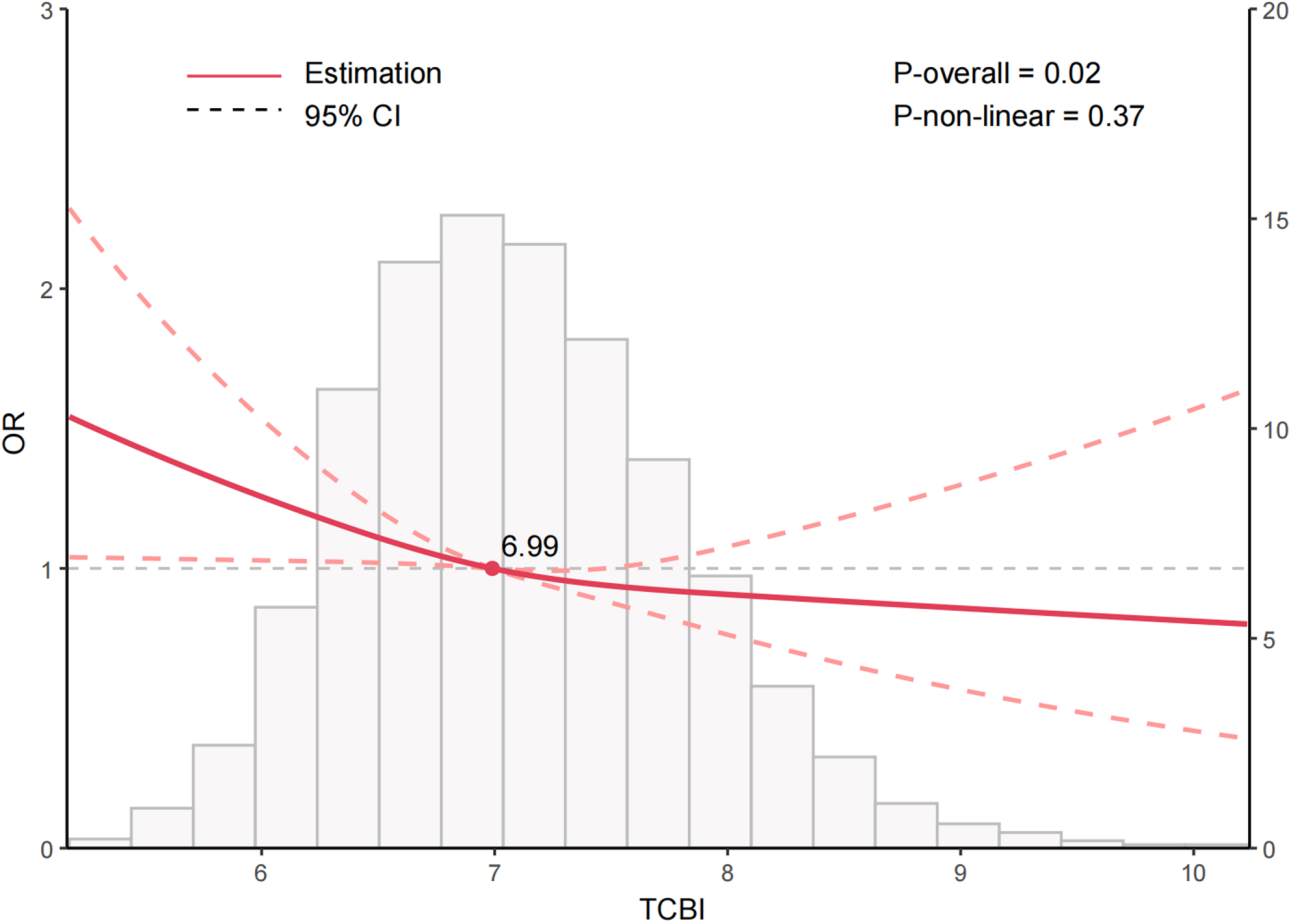
Nonlinear relationship between TCBI and edentulism in the cross-sectional study

As shown in Table 2, when treated as a continuous measure, TCBI displayed a significant inverse association with edentulism in the unadjusted model (OR = 0.69, 95% CI: 0.62–0.77, *P* < 0.001). This inverse association persisted after stepwise adjustment for covariates (OR = 0.86, 95% CI: 0.76–0.96, *P* = 0.009). In the tertile analysis, individuals in the highest tertile (T3) exhibited a significantly lower risk of edentulism than those in the lowest tertile (T1) (OR = 0.80, 95% CI: 0.65–0.97, *P* = 0.03). A test for trend indicated a decreasing risk of edentulism with increasing TCBI levels (*P* for trend = 0.03). RCS modeling further confirmed this linear inverse association (*P* overall = 0.02), and no significant non-linear effect was observed (*P* non-linear = 0.37) (Fig. 2).

**Table 2:** Association between TCBI and edentulism in the cross-sectional study

Characteristic	Model 1 OR (95% CI) ^*^ *P* value	Model 2 OR (95% CI) ^*^ *P* value	Model 3 OR (95% CI) ^*^ *P* value
**TCBI** **(Continuous)**	0.69 (0.62, 0.77) < 0.001	0.83 (0.74, 0.93) < 0.001	0.86 (0.76, 0.96) 0.009
**TCBI tertiles**			
T1 (3.19–6.75)	Ref	Ref	Ref
T2 (6.75–7.37)	0.80 (0.68, 0.94) 0.008	0.88 (0.74, 1.05) 0.15	0.90 (0.75, 1.08) 0.24
T3 (7.37–11.33)	0.57 (0.48, 0.68) < 0.001	0.76 (0.62, 0.92) 0.005	0.80 (0.65, 0.97) 0.03
***P*** **for trend**	< 0.001	0.002	0.03

Model 1: Non-adjusted

Model 2: Adjusted for age, sex, education, marital status, smoke, drinking, residence

Model 3: Further adjusted for hypertension and diabetes based on model 2

TCBI values were log-transformed

^*^OR Odds Ratio, *CI* Confidence Interval

### Prospective cohort analysis: baseline characteristics

According to Tables 3, 8,568 participants were included in the prospective cohort and stratified into tertiles based on their TCBI levels: T1 (*n* = 2,860), T2 (*n* = 2,852), and T3 (*n* = 2,856). Age distribution differed significantly across TCBI tertiles, with median ages of 58.00 years in T1, 58.00 years in T2, and 57.00 years in T3 (*P* < 0.001). Sex distribution also varied, with the highest proportion of females in T3 (56.41%) and a relatively lower proportion in T1 (50.49%, *P* < 0.001). Educational attainment differed across TCBI tertiles. Participants without formal education were most common in T1 (49.30%) and became progressively fewer in T2 (45.65%) and T3 (42.05%) (*P* < 0.001). Residence also differed significantly across tertiles, with rural proportions decreasing from 70.17% (T1) to 65.15% (T2) and 59.00% (T3), while urban proportions increased accordingly (*P* < 0.001). Regarding marital status, the proportion of married participants was highest in T3 (91.74%) and lowest in T1 (88.08%, *P* < 0.001). For lifestyle characteristics, both smoking prevalence (*P* < 0.001) and drinking prevalence (*P* = 0.04) declined with increasing TCBI. In terms of clinical conditions, the prevalence of hypertension was highest in T3 (51.79%) and lowest in T1 (28.85%, *P* < 0.001). Similarly, diabetes prevalence increased across groups (3.39% vs. 5.26% vs. 8.93%, *P* < 0.001). The prevalence of edentulism was highest in T1 (16.22%) and lowest in T3 (11.76%, *P* < 0.001).

**Table 3:** Baseline characteristics in the prospective cohort study

Characteristic	T1 (3.30–6.77) *n* = 2,860	T2 (6.77–7.39) *n* = 2,852	T3 (7.39–11.33) *n* = 2,856	*P* Value
**Age**	58.00 [51.00;65.00]	58.00 [51.00;64.00]	57.00 [51.00;63.00]	< 0.001
**Sex**				< 0.001
Female	1444 (50.49%)	1592 (55.82%)	1611 (56.41%)	
Male	1416 (49.51%)	1260 (44.18%)	1245 (43.59%)	
**Education**				< 0.001
College or above	32 (1.12%)	43 (1.51%)	42 (1.47%)	
Middle school	765 (26.75%)	822 (28.82%)	969 (33.93%)	
No formal education	1410 (49.30%)	1302 (45.65%)	1201 (42.05%)	
Primary school	653 (22.83%)	685 (24.02%)	644 (22.55%)	
**Residence**				< 0.001
Rural	2007 (70.17%)	1858 (65.15%)	1685 (59.00%)	
Urban	853 (29.83%)	994 (34.85%)	1171 (41.00%)	
**Marital status**				< 0.001
Married	2519 (88.08%)	2555 (89.59%)	2620 (91.74%)	
Others	313 (10.94%)	284 (9.96%)	225 (7.88%)	
Unmarried	28 (0.98%)	13 (0.46%)	11 (0.39%)	
**Smoke**				< 0.001
No	1675 (58.57%)	1817 (63.71%)	1810 (63.38%)	
Yes	1185 (41.43%)	1035 (36.29%)	1046 (36.62%)	
**Drinking**				0.04
No	1873 (65.49%)	1924 (67.46%)	1959 (68.59%)	
Yes	987 (34.51%)	928 (32.54%)	897 (31.41%)	
**Hypertension**				< 0.001
No	2035 (71.15%)	1746 (61.22%)	1377 (48.21%)	
Yes	825 (28.85%)	1106 (38.78%)	1479 (51.79%)	
**Diabetes**				< 0.001
No	2763 (96.61%)	2702 (94.74%)	2601 (91.07%)	
Yes	97 (3.39%)	150 (5.26%)	255 (8.93%)	
**Edentulism**				< 0.001
No	2396 (83.78%)	2442 (85.62%)	2520 (88.24%)	
Yes	464 (16.22%)	410 (14.38%)	336 (11.76%)	

### Prospective cohort analysis: association between TCBI and incident edentulism

**Fig. 3:**
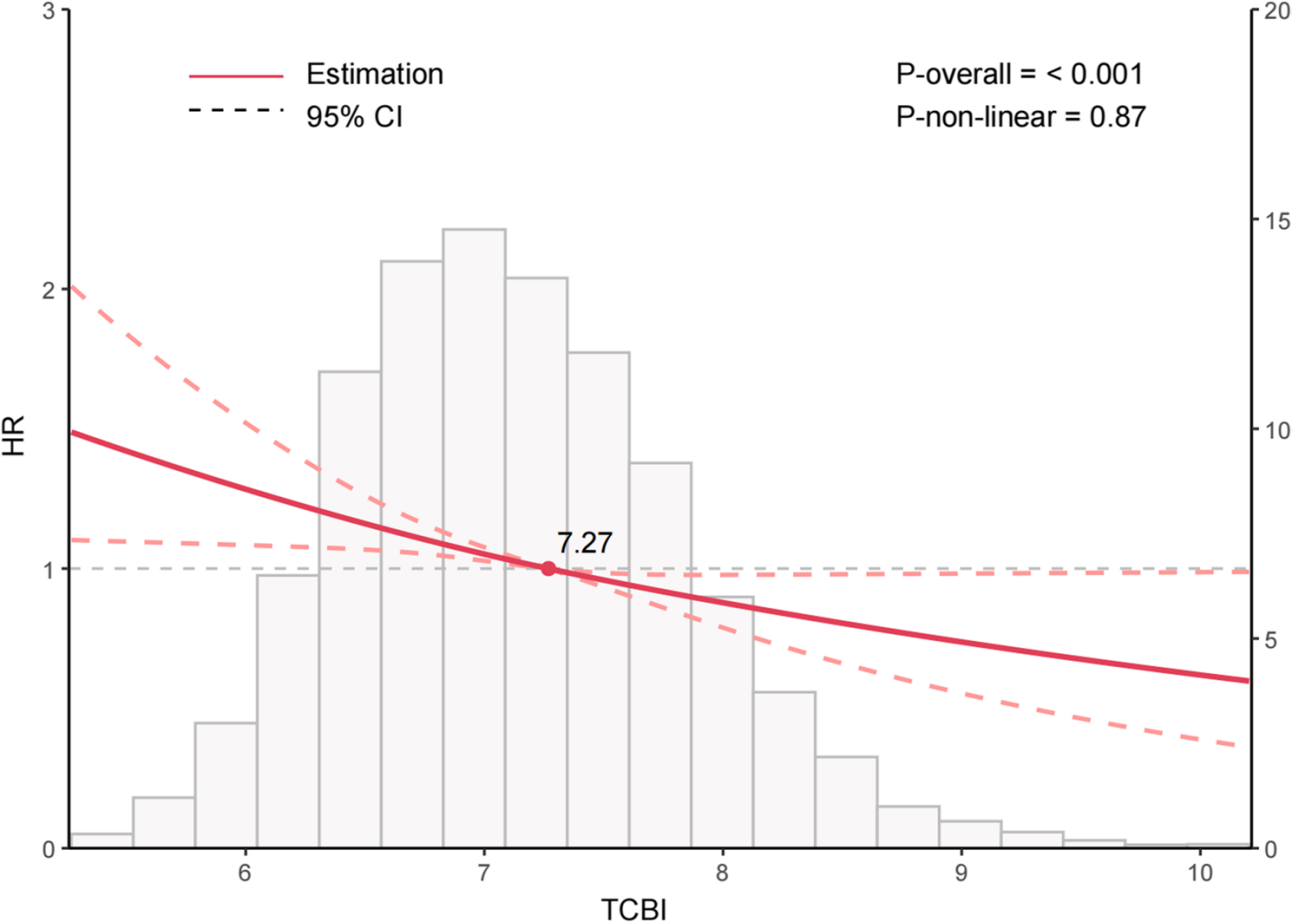
Nonlinear relationship between TCBI and edentulism in the prospective cohort study

In the prospective cohort, higher TCBI levels were significantly associated with a reduced risk of edentulism (Table 4). When analyzed as a continuous variable, the unadjusted model showed that each unit increase in TCBI was associated with a significantly lower risk of edentulism (HR = 0.77, 95% CI: 0.71–0.84, *P* < 0.001). This inverse association persisted after stepwise adjustment for demographic factors, lifestyle behaviors, and comorbidities (fully adjusted HR = 0.85, 95% CI: 0.77–0.92, *P* < 0.001). In the tertile analysis, participants in the highest TCBI tertile (T3) demonstrated a significantly lower risk of edentulism relative to those in the lowest tertile (T1) (HR = 0.82, 95% CI: 0.71–0.95, *P* = 0.009). A test for trend indicated a significant decreasing risk of edentulism with increasing TCBI levels (*P* for trend = 0.009). RCS modeling further demonstrated an approximately linear relationship between TCBI and edentulism risk (*P* overall < 0.001), with no detectable evidence of non-linearity (*P* non-linear = 0.87) (Fig. 3).

**Table 4:** Association between TCBI and edentulism in the prospective cohort study

Characteristic	Model 1 HR (95% CI) ^1^ *P* value	Model 2 HR (95% CI) ^1^ *P* value	Model 3 HR (95% CI) ^1^ *P* value
**TCBI** **(Continuous)**	0.77 (0.71, 0.84) < 0.001	0.85 (0.78, 0.92) < 0.001	0.85 (0.77, 0.92) < 0.001
**TCBI tertiles**			
T1	Ref	Ref	Ref
T2	0.87 (0.76, 0.99) 0.04	0.92 (0.80, 1.05) 0.20	0.92 (0.82, 1.05) 0.21
T3	0.70 (0.61, 0.81) < 0.001	0.82 (0.71, 0.94) 0.006	0.82 (0.71, 0.95) 0.009
***P*** **for trend**	< 0.001	0.006	0.009

Model 1: Non-adjusted

Model 2: Adjusted for age, sex, education, marital status, smoke, drinking, residence

Model 3: Further adjusted for hypertension and diabetes based on model 2

TCBI values were log-transformed

^1^*HR* Hazard Ratio, *CI* Confidence Interval

### Trajectory analysis: baseline characteristics

**Fig. 4:**
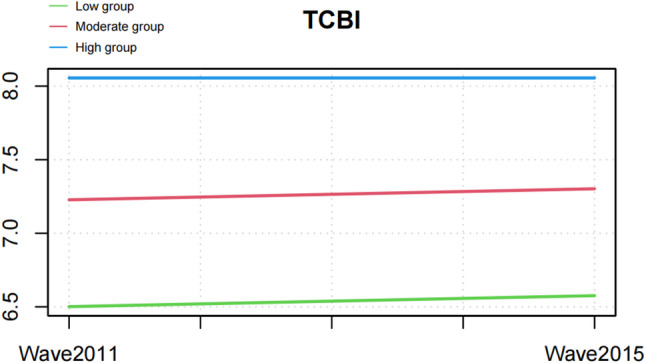
Longitudinal trajectories of TCBI identified by group-based trajectory modeling

In the trajectory analysis cohort, 4,921 participants were included and subsequently classified into the low-TCBI group (*n* = 1,639), moderate-TCBI group (*n* = 2,461), and high-TCBI group (*n* = 821) (Fig. 4; Table 5). With increasing TCBI levels, participants were younger and the proportion of females increased, with median ages of 58.00, 57.00, and 56.00 years in the low-, moderate-, and high-TCBI groups, respectively (*P* < 0.001). The proportion of females was highest in the high-TCBI group (59.56%, *P* < 0.001). Educational attainment also varied significantly, with a higher proportion of middle school or higher education in the high-TCBI group, whereas nearly half of participants in the low-TCBI group had no formal education (49.91%, *P* < 0.001). Residence also differed across groups, with the proportion of rural residents highest in the low-TCBI group (71.81%) and lowest in the high-TCBI group (56.64%) (*P* < 0.001). In addition, the proportion of married individuals was highest in the high-TCBI group (94.40%, *P* < 0.001), and the prevalence of smoking declined progressively with increasing TCBI (34.59%, *P* < 0.001). Differences in drinking status were relatively small, with only a slightly higher prevalence in the low-TCBI group compared with the others (35.02%, *P* = 0.04). Regarding clinical characteristics, the prevalence of hypertension (53.35%, *P* < 0.001) and diabetes (10.48%, *P* < 0.001) was significantly higher in the high-TCBI group. Moreover, TCBI values measured in both 2011 and 2015 increased progressively across the groups (both *P* < 0.001), while the prevalence of edentulism decreased gradually with increasing TCBI (*P* < 0.001) (Table 5).

**Table 5:** Baseline characteristics in the trajectory analysis cohort

	Low-TCBI group (*n* = 1,639)	Moderate-TCBI group (*n* = 2,461)	High-TCBI group (*n* = 821)	*P* value
**Age**	58.00 [51.00;64.00]	57.00 [51.00;62.00]	56.00 [50.00;60.00]	< 0.001
**Sex**:				< 0.001
Female	827 (50.46%)	1447 (58.80%)	489 (59.56%)	
Male	812 (49.54%)	1014 (41.20%)	332 (40.44%)	
**Education**:				< 0.001
College or above	17 (1.04%)	34 (1.38%)	11 (1.34%)	
Middle school	438 (26.72%)	784 (31.86%)	283 (34.47%)	
No formal education	818 (49.91%)	1056 (42.91%)	327 (39.83%)	
Primary school	366 (22.33%)	587 (23.85%)	200 (24.36%)	
**Residence**:				< 0.001
Rural	1177 (71.81%)	1602 (65.10%)	465 (56.64%)	
Urban	462 (28.19%)	859 (34.90%)	356 (43.36%)	
**Marital status**:				< 0.001
Married	1467 (89.51%)	2255 (91.63%)	775 (94.40%)	
Others	155 (9.46%)	201 (8.17%)	45 (5.48%)	
Unmarried	17 (1.04%)	5 (0.20%)	1 (0.12%)	
**Smoke**:				< 0.001
No	976 (59.55%)	1618 (65.75%)	537 (65.41%)	
Yes	663 (40.45%)	843 (34.25%)	284 (34.59%)	
**Drinking**:				0.04
No	1065 (64.98%)	1690 (68.67%)	557 (67.84%)	
Yes	574 (35.02%)	771 (31.33%)	264 (32.16%)	
**Hypertension**:				< 0.001
No	1182 (72.12%)	1455 (59.12%)	383 (46.65%)	
Yes	457 (27.88%)	1006 (40.88%)	438 (53.35%)	
**Diabetes**:				< 0.001
No	1583 (96.58%)	2329 (94.64%)	735 (89.52%)	
Yes	56 (3.42%)	132 (5.36%)	86 (10.48%)	
**TCBI wave2011**	6.49 [6.26;6.72]	7.24 [6.97;7.53]	8.13 [7.82;8.46]	< 0.001
**TCBI wave2015**	6.55 [6.32;6.77]	7.32 [7.06;7.59]	8.19 [7.89;8.52]	< 0.001
**Edentulism**:				< 0.001
No	1512 (92.25%)	2321 (94.31%)	790 (96.22%)	
Yes	127 (7.75%)	140 (5.69%)	31 (3.78%)	

### Trajectory analysis: association between TCBI trajectories and edentulism risk

In the trajectory analysis cohort, participants with higher TCBI levels had a significantly lower risk of edentulism (Table 6). Compared with the low-TCBI group, the moderate-TCBI group showed a lower risk in the unadjusted model (HR = 0.73, 95% CI: 0.57–0.92, *P* = 0.009); however, this association was attenuated and lost statistical significance after stepwise adjustment for demographic characteristics and lifestyle factors (fully adjusted HR = 0.80, 95% CI: 0.63–1.02, *P* = 0.07). In contrast, the high-TCBI group consistently demonstrated a protective effect across models, with an unadjusted HR of 0.48 (95% CI: 0.32–0.71, *P* < 0.001) and a fully adjusted HR of 0.59 (95% CI: 0.40–0.89, *P* = 0.01). These findings suggest that a robust reduction in edentulism risk was observed only at higher TCBI levels.

**Table 6:** Association between TCBI and edentulism in the trajectory analysis cohort

Characteristic	Model 1 HR (95% CI) ^*^ P value	Model 2 HR (95% CI) ^*^ P value	Model 3 HR (95% CI) ^*^ P value
Low-TCBI group	Ref	Ref	Ref
Moderate-TCBI group	0.73 (0.57, 0.92) 0.009	0.81 (0.63, 1.03) 0.08	0.80 (0.63, 1.02) 0.07
High-TCBI group	0.48 (0.32, 0.71) < 0.001	0.60 (0.41, 0.90) 0.01	0.59 (0.40, 0.89) 0.01

TCBI values were log-transformed

Model 1: Non-adjusted

Model 2: Adjusted for age, sex, education, marital status, smoke, drinking, residence

Model 3: Further adjusted for hypertension and diabetes based on model 2

^*^ *HR* Hazard Ratio, *CI* Confidence Interval

### Sensitivity analyses

In the sensitivity analyses, participants were categorized according to cumulative TCBI levels. The results showed that the incidence of edentulism decreased progressively with higher cumulative TCBI levels (Supplementary Table 5, *P* < 0.001). Multivariable regression further confirmed that higher cumulative TCBI levels were independently associated with a lower risk of edentulism (HR = 0.67, 95% CI: 0.50–0.91, *P* = 0.01). These results aligned with the primary analyses, suggesting that the conclusions were robust (Supplementary Table 6).

## Discussion

Using data from a large, nationally representative cohort, this study provides the first comprehensive evaluation of TCBI in relation to edentulism across cross-sectional, prospective, and trajectory analyses. Higher TCBI levels were consistently associated with a lower risk of edentulism, and individuals maintaining higher TCBI over time exhibited the greatest protection. RCS analyses further indicated a monotonic decrease in the probability of edentulism with increasing TCBI levels. These findings suggest that TCBI, a simple and cost-effective nutritional index derived from routine clinical parameters, may serve as a practical tool for identifying individuals at high risk of edentulism and for guiding early nutrition-based preventive strategies in clinical and public health practice.

Existing reviews and population-based studies have consistently demonstrated a stable association between nutritional status and edentulism. Multiple systematic reviews in older adults have reported that individuals with partial or complete edentulism exhibit a higher prevalence of malnutrition or nutritional risk. Although heterogeneity exists among studies in terms of assessment methods and adjustment for confounding variables, the overall direction of association remains consistent [9]. Prospective cohort studies have also shown that individuals with reduced dentition often experience decreased dietary diversity and poorer diet quality, both of which are closely linked to insufficient intake of energy, protein, and micronutrients [30, 31]. These findings suggest a close interrelationship between nutritional status and changes in dentition. Broader epidemiological evidence has further indicated that edentulism is strongly associated with malnutrition and a decline in overall health status [32], implying that long-term nutritional inadequacy may play a crucial role in the progressive loss of teeth leading to complete edentulism.

Building upon this foundation, the present study adds to existing evidence by applying a multidimensional analytical framework. The consistent inverse association observed across cross-sectional, longitudinal, and trajectory analyses strengthens the evidential credibility of the findings and helps mitigate the temporal constraints inherent to traditional cross-sectional designs. Notably, the trajectory analysis revealed that sustained declines in TCBI were associated with the highest risk of edentulism, offering new evidence for the cumulative impact of long-term nutritional insufficiency on oral health. Compared with earlier studies focusing on single nutrient indicators or self-reported dietary habits, TCBI—derived from objective clinical measurements—provides a more comprehensive and reproducible reflection of overall nutritional reserves.

Lifestyle behaviors—including dietary patterns, smoking, alcohol consumption, and physical activity—have been widely examined in relation to tooth loss; however, their explanatory power at the population level remains limited. These behaviors are subject to socioeconomic, cultural, and adherence-related variability, and inconsistencies in measurement methods have led to poor reproducibility [33–35]. Recent systematic reviews have also highlighted considerable methodological heterogeneity and limited certainty of evidence regarding the relationship between health behaviors and tooth loss [35]. In contrast, TCBI offers several methodological advantages, including objectivity, standardization, quantifiability, and accessibility. Multiple studies across diverse disease domains have validated its clinical relevance, showing that lower TCBI levels are strongly associated with higher risks of cardiovascular events, adverse cancer outcomes, and all-cause mortality [36–38]. Collectively, these findings indicate that TCBI provides a more comprehensive reflection of overall nutritional reserves and demonstrates strong reproducibility and external applicability.

The clinical and public health significance of this study lies in the potential of TCBI, a composite nutritional index derived from routine clinical parameters, to serve as a simple, objective, and cost-effective tool for oral health risk assessment. Its integration into primary care and community screening programs could enable earlier recognition of people likely to be at elevated risk of edentulism and provide an evidence-based framework for nutritional intervention and personalized health management.

### Strengths and limitations

This study has several notable strengths. Leveraging CHARLS, a large and nationally representative cohort, increased the population-level relevance and generalizability of the findings. The multidimensional analytical framework, which incorporated cross-sectional, prospective, and trajectory analyses, provided a rigorous and comprehensive evaluation of both static and dynamic nutritional effects on edentulism. The use of TCBI as an objective and reproducible nutritional index derived from routine clinical measurements improved measurement accuracy compared with self-reported dietary data. In addition, advanced statistical techniques, including RCS and trajectory modeling, enabled precise assessment of dose–response relationships and long-term nutritional patterns.

Despite the large sample size and multidimensional design, certain study constraints remain. First, although multiple covariates were adjusted for, the possibility of residual confounding cannot be fully excluded. For instance, the CHARLS database does not include information on oral hygiene practices or oral health behaviors that may influence tooth loss, and these variables could play important roles in the development of edentulism. The absence of such data may have introduced unmeasured confounding, potentially affecting the stability and broader applicability of the results. Second, edentulism status was determined based on self-reported information rather than clinical examination, and detailed data on the number of remaining teeth or periodontal condition were unavailable. This limitation may have introduced minor measurement bias; nevertheless, prior research assessing the accuracy of self-reported oral conditions has indicated that older adults’ reports of tooth loss correspond well with outcomes obtained from clinical evaluations [24, 25]. Third, because the analysis was based on a Chinese cohort, the external validity of the findings should be interpreted with caution.

Additional investigations are needed to corroborate the present results in heterogeneous populations and incorporate clinical oral examinations, objective dental records, and oral hygiene behavior data. In addition, prospective interventional studies are warranted to determine whether improving nutritional status and increasing TCBI levels can reduce the risk of edentulism, thereby providing direct evidence to inform prevention and management strategies.

## Conclusions

Higher TCBI levels were consistently associated with a lower risk of edentulism across cross-sectional, prospective, and trajectory analyses. As a simple and objective nutritional indicator, TCBI may help identify individuals with poor nutritional reserves who are at increased risk of tooth loss and may support earlier preventive dental care. Further studies, including interventional research and validation in diverse populations, are needed to determine whether improving nutritional status or modifying TCBI levels can slow or prevent the progression of edentulism.

## Supplementary Information

Supplementary Material 1. Supplementary Figure 1

Supplementary Material 2. Supplementary Figure 2

Supplementary Material 3. Language Editing Certificate-FQHFX

Supplementary Material 4. iThenticate Similarity Report

Supplementary Material 5. Supplementary Tables

## Data Availability

This study relied on de-identified information provided by the China Health and Retirement Longitudinal Study (CHARLS). Access to the CHARLS dataset can be requested by eligible researchers through the official application portal (http://charls.pku.edu.cn) after completing the required registration procedures. Requests for research materials related to this work may be directed to the corresponding author, who will provide access when the inquiry is considered appropriate.

## Abbreviations

TCBI: triglyceride–cholesterol–body weight index
CHARLS: China Health and Retirement Longitudinal Study
RCS: restricted cubic spline
TG: triglyceride
OR: odds ratio
BMI: body mass index
TC: total cholesterol
GBTM: group-based trajectory modeling
CI: confidence interval
HR: hazard ratio
DAG: directed acyclic graph
GVIF: generalized variance inflation factor

## References

[1] Tang Z, Huang C, Li Y, Sun Y, Chen X. Early-life adversity and edentulism among Chinese older adults. BMC Oral Health. 2022;22(1):542. 36434640 10.1186/s12903-022-02595-3 PMC9700936

[2] Huraib WM, Al-Ghalib TA, Niyazi AAT, Bamigdad MS. Assessment of nutritional and psychosocial status of elderly patients wearing removable dental prosthetics. J Pharm Bioallied Sci. 2022;14(Suppl 1):S429–32. 36110758 10.4103/jpbs.jpbs_840_21 PMC9469388

[3] Ijaopo E, Ijaopo R. A review of oral health in older adults: key to improving nutrition and quality of life. OBM Geriatr. 2018;2(3):1–35.

[4] Zhang XM, Cao S, Teng L, Xie X, Wu X. The association between the number of teeth and frailty among older adults: a systematic review and meta-analysis. Aging Clin Exp Res. 2025;37(1):156. 40377807 10.1007/s40520-025-03053-0 PMC12084268

[5] Li Y, Guo M, Fei Y, Liu Y, AL-Ghammari A, Chen S, et al. Association between oral health and physio-cognitive decline syndrome of older adults in China and its sex differences: a cross-sectional study. BMC Geriatr. 2025;25(1):137. 40021985 10.1186/s12877-025-05801-3 PMC11871684

[6] Aminoshariae A, Nosrat A, Jakovljevic A, Jaćimović J, Narasimhan S, Nagendrababu V. Tooth loss is a risk factor for cardiovascular disease mortality: A systematic review with Meta-analyses. J Endod. 2024;50(10):1370–80. 38945200 10.1016/j.joen.2024.06.012

[7] Al-Rafee MA. The epidemiology of edentulism and the associated factors: A literature review. J Family Med Prim Care. 2020;9(4):1841. 32670928 10.4103/jfmpc.jfmpc_1181_19 PMC7346915

[8] Jordan AR, Stark H, Nitschke I, Micheelis W, Schwendicke F. Epidemiological trends, predictive factors, and projection of tooth loss in Germany 1997–2030: part I. missing teeth in adults and seniors. Clin Oral Invest. 2021;25(1):67–76. 10.1007/s00784-020-03266-9 PMC7785540 33219875

[9] Kaurani P, Kakodkar P, Bhowmick A, Samra RK, Bansal V. Association of tooth loss and nutritional status in adults: an overview of systematic reviews. BMC Oral Health. 2024;24:838. 39049002 10.1186/s12903-024-04602-1 PMC11267674

[10] Berg Y, Gabay E, Božić D, Shibli JA, Ginesin O, Asbi T, et al. The impact of nutritional components on periodontal health: A literature review. Nutrients. 2024;16(22):3901. 39599688 10.3390/nu16223901 PMC11597335

[11] Dommisch H, Kuzmanova D, Jönsson D, Grant M, Chapple I. Effect of micronutrient malnutrition on periodontal disease and periodontal therapy. Periodontol 2000. 2018;78(1):129–53. 30198127 10.1111/prd.12233

[12] Seth I, Lim B, Cevik J, Gracias D, Chua M, Kenney PS, et al. Impact of nutrition on skin wound healing and aesthetic outcomes: A comprehensive narrative review. JPRAS Open. 2024;39:291–302. 38370002 10.1016/j.jpra.2024.01.006 PMC10874171

[13] Algra Y, Haverkort E, Kok W, van Etten-Jamaludin F, van Hollaar SL. The association between malnutrition and oral health in older people: A systematic review. Nutrients. 2021;13(10):3584. 34684584 10.3390/nu13103584 PMC8541038

[14] Toniazzo MP, Amorim P, de Muniz S, Weidlich FWMG. P. Relationship of nutritional status and oral health in elderly: Systematic review with meta-analysis. Clin Nutr. 2018;37(3):824–30. 28392164 10.1016/j.clnu.2017.03.014

[15] Yang B, Yang Y, Liu B, Yang M. Role of composite objective nutritional indexes in patients with chronic kidney disease. Front Nutr. 2024;11:1349876. 38699544 10.3389/fnut.2024.1349876 PMC11063252

[16] Bhattacharya A, Pal B, Mukherjee S, Roy SK. Assessment of nutritional status using anthropometric variables by multivariate analysis. BMC Public Health. 2019;19(1):1045. 31382936 10.1186/s12889-019-7372-2 PMC6683359

[17] Liu G, Zhang J. Association of a novel nutritional index with cognitive impairment in middle-aged and elderly Chinese adults: a cross-sectional analysis from the China health and retirement longitudinal study. Front Nutr. 2025;12:1486917. 39963661 10.3389/fnut.2025.1486917 PMC11830621

[18] Doi S, Iwata H, Wada H, Funamizu T, Shitara J, Endo H, et al. A novel and simply calculated nutritional index serves as a useful prognostic indicator in patients with coronary artery disease. Int J Cardiol. 2018;262:92–8. 29706396 10.1016/j.ijcard.2018.02.039

[19] Słotwińska SM, Słotwiński R. Host response, malnutrition and oral diseases. Part 1. Cent Eur J Immunol. 2014;39(4):518–21. 26155172 10.5114/ceji.2014.47738 PMC4439965

[20] Wang S, Wang Y, Yu R, Yuan D, Ni Y, Wang L, et al. Association of lipid profile and reported edentulism in the elder population: data from the China health and retirement longitudinal study. BMC Oral Health. 2022;22(1):445. 36243707 10.1186/s12903-022-02492-9 PMC9571461

[21] Guo M, Zhang Z, Dong H, Liu L, Zhao Z, Li X, et al. Association between the psychological frailty index and stroke: a cohort study from CHARLS. Sci Rep. 2025;15:29756. 40804454 10.1038/s41598-025-15270-8 PMC12350624

[22] Luwen H, Yunwei Z, Lei X, Linlin L, Ming Y. Unraveling the role of cumulative triglyceride-total cholesterol-body weight index in stroke development: evidence from the CHARLS cohort. Front Med (Lausanne). 2025;10:12. 10.3389/fmed.2025.1616520 PMC12287075 40708645

[23] Wu D, Mao M, Wang W, Zheng H, You H, Chen W, et al. Tooth loss and mortality risk: the mediating role of hs-CRP in a Chinese cohort. Front Oral Health. 2025;6:1542147. 40342577 10.3389/froh.2025.1542147 PMC12058789

[24] Høvik H, Kolberg M, Gjøra L, Nymoen LC, Skudutyte-Rysstad R, Hove LH, et al. The validity of self-reported number of teeth and edentulousness among Norwegian older adults, the HUNT study. BMC Oral Health. 2022;22:82. 35313882 10.1186/s12903-022-02116-2 PMC8935783

[25] Shimazaki Y, Saito M, Nonoyama T, Inamoto Y. Validity of the self-reported number of teeth in independent older people in Japan. BMC Geriatr. 2024;24(1):900. 39482622 10.1186/s12877-024-05512-1 PMC11526520

[26] Chen X, Zeng C, Chen X, Sun J, Li Y, Chen Z, et al. The impact of physical activity on the prevalence of edentulism: an analysis of the relationships between active lifestyle and dental health. BMC Public Health. 2024;24:2743. 39379907 10.1186/s12889-024-20242-0 PMC11462750

[27] Madlock-Brown C, Wilkens K, Weiskopf N, Cesare N, Bhattacharyya S, Riches NO, et al. Clinical, social, and policy factors in COVID-19 cases and deaths: methodological considerations for feature selection and modeling in county-level analyses. BMC Public Health. 2022;22:747. 35421958 10.1186/s12889-022-13168-y PMC9008430

[28] Nguena Nguefack HL, Pagé MG, Katz J, Choinière M, Vanasse A, Dorais M, et al. Trajectory modelling techniques useful to epidemiological research: A comparative narrative review of approaches. Clin Epidemiol. 2020;12:1205–22. 33154677 10.2147/CLEP.S265287 PMC7608582

[29] Nagin DS, Odgers CL. Group-Based trajectory modeling in clinical research. Annu Rev Clin Psychol. 2010;6(1):109–38. 20192788 10.1146/annurev.clinpsy.121208.131413

[30] Winning L, Logan D, McEvoy CT, Farsi D, McKay GJ, Patterson CC, et al. Tooth loss, diet quality, and cognitive decline: A 15-year longitudinal study. J Nutr Health Aging. 2025;29(9):100620. 40580824 10.1016/j.jnha.2025.100620 PMC12268072

[31] Xu X, Zhao Y, Wu B, Pei Y, Gu D. Association between tooth loss and frailty among Chinese older adults: the mediating role of dietary diversity. BMC Geriatr. 2023;23(1):668. 37848821 10.1186/s12877-023-04355-6 PMC10583397

[32] Iacob S, Chisnoiu RM, Zaharia A, Bălaj MG, Iosa AE, Condor AM, et al. Correlation between type of Edentulism, Age, socioeconomic status and general health. J Clin Med. 2025;14(11):3924. 40507685 10.3390/jcm14113924 PMC12155793

[33] dos Anjos SD, Ferro RM, Laskawski BN, Haas AN, Prates RC, Steffens JP. Associations between physical activity domains and oral health: an analysis of a Brazilian population–based study. Braz Oral Res. 2023;37:e071. 37436294 10.1590/1807-3107bor-2023.vol37.0071

[34] Gomez GGF, Xu H, Spence LA, Patel P, Bualteng V, Cheriyan B, et al. Assessing the nutrient intake and diet quality of adults wearing dentures using the healthy eating index. BMC Oral Health. 2025;25:996. 10.1186/s12903-025-06182-0 PMC12220130 40604813

[35] Alobaidi F, Heidari E, Sabbah W. Systematic review of longitudinal studies on the association between cluster of health-related behaviors and tooth loss among adults. Acta Odontol Scand. 2024;83:40284. 38014435 10.1080/00016357.2023.2287718 PMC11302646

[36] Rezaee M, Kamrani F, Imannezhad M, Shahri HH, Saihood WK, Rezvani A, et al. Beyond traditional metrics: evaluating the triglyceride-total cholesterol-body weight index (TCBI) in cardiovascular risk assessment. BMC Cardiovasc Disord. 2025;25(1):39. 39849378 10.1186/s12872-025-04500-6 PMC11756170

[37] Imannezhad M, Kamrani F, Shariatikia A, Nasrollahi M, Mahaki H, Rezaee A, et al. Association of atherogenic indices and triglyceride-total cholesterol-body weight index (TCBI) with severity of stenosis in patients undergoing angiography: a case-control study. BMC Res Notes. 2025;18:180. 40247425 10.1186/s13104-025-07203-5 PMC12004764

[38] Li G, Li S. Exploring the prognostic value of the novel nutritional index for in-hospital mortality in acute coronary syndrome: a sex-specific analysis. Front Med (Lausanne). 2025;12:1498260. 40342584 10.3389/fmed.2025.1498260 PMC12058770

