# Supplementary figures and images for: A novel nutritional index and risk of edentulism: evidence from cross-sectional, prospective, and trajectory analyses
Source: Lipids Health Dis. 2026 Jan 14;25:50. doi: 10.1186/s12944-026-02860-2 (PMC12888110; doi:10.1186/s12944-026-02860-2)

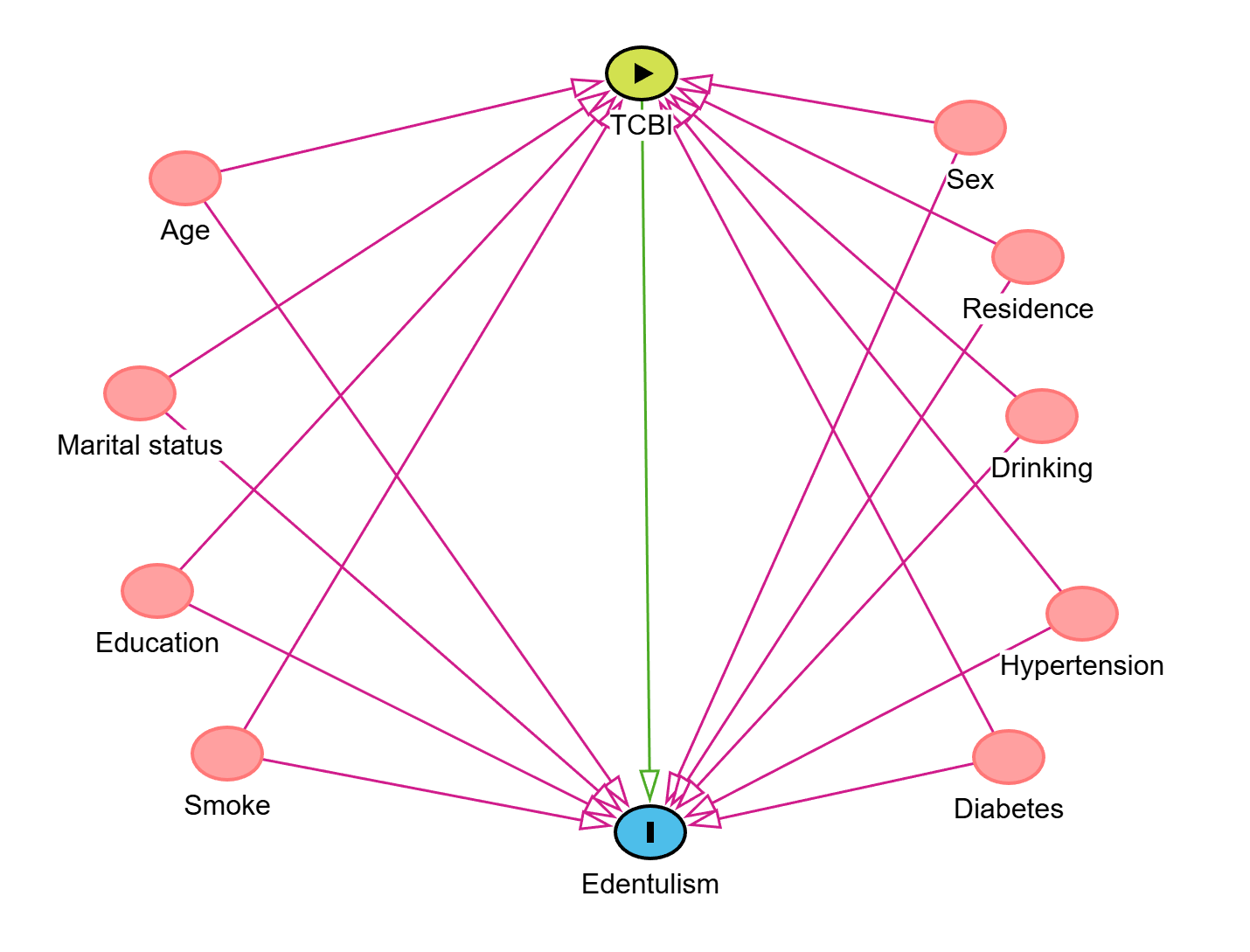

Supplement: Supplementary file 1 — Supplementary Material 1. Supplementary Figure 1 [file 12944_2026_2860_MOESM1_ESM.tif]

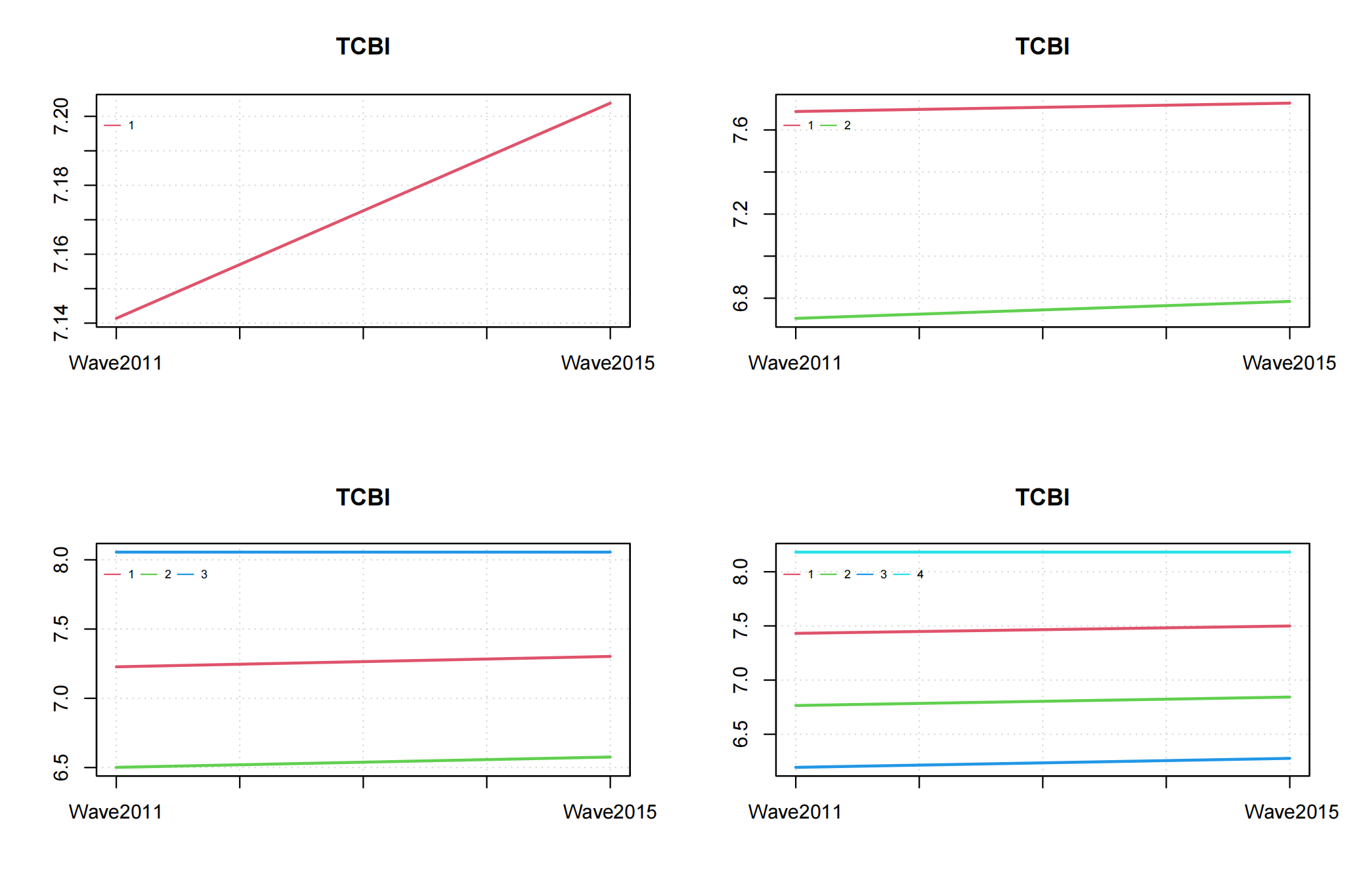

Supplement: Supplementary file 2 — Supplementary Material 2. Supplementary Figure 2 [file 12944_2026_2860_MOESM2_ESM.tif]
