# Supplementary material for: A novel nutritional index and risk of edentulism: evidence from cross-sectional, prospective, and trajectory analyses
Source: Lipids Health Dis. 2026 Jan 14;25:50. doi: 10.1186/s12944-026-02860-2 (PMC12888110; doi:10.1186/s12944-026-02860-2)
Supplement: Supplementary file 3 — Supplementary Material 3. Language Editing Certificate-FQHFX [file 12944_2026_2860_MOESM3_ESM.pdf]

Editing Certificate

This document certifies that the manuscript listed below has been edited to ensure language and grammar accuracy and is error free in these aspects. The edit was performed by professional editors at Editage, a brand of Cactus Communications. The author's core research ideas were not altered in any way during the editing process. The quality of the edit has been guaranteed, with the assumption that our suggested changes have been accepted and the text has not been further altered without the knowledge of our editors.

MANUSCRIPT TITLE

A Novel Nutritional Index and Risk of Edentulism: Evidence from Cross-Sectional, Prospective, and Trajectory Analyses

AUTHORS

Cao Yue

ISSUED ON

December 16, 2025

JOB CODE

FQHFX_2

Prabh Grewal
Senior Vice President - Editage

editage | helping you get published

Since 2002, Editage has helped over 430,000 authors publish around 1.2 million research papers in scholarly journals across over 1000 disciplines through editorial, translation, transcription, and publication support services. Editage is a brand of Cactus Communications (cactusglobal.com), a science communication and technology company.

CACTUS.

GLOBAL :
+1(669) 272-1214 | request@editage.com

CHINA :
400-001-8237; 021-60209400 | fabiao@editage.cn
