# Supplementary material for: A novel nutritional index and risk of edentulism: evidence from cross-sectional, prospective, and trajectory analyses
Source: Lipids Health Dis. 2026 Jan 14;25:50. doi: 10.1186/s12944-026-02860-2 (PMC12888110; doi:10.1186/s12944-026-02860-2)
Supplement: Supplementary file 4 — Supplementary Material 4. iThenticate Similarity Report [file 12944_2026_2860_MOESM4_ESM.pdf]

20251218013750119884367412281753

WORD COUNT	4222	TIME SUBMITTED	18-DEC-2025 01:37AM
		PAPER ID	119531992

A Novel Nutritional Index and Risk of Edentulism: Evidence from Cross-Sectional, Prospective, and Trajectory Analyses

Qi Luo [1], Qian Yang [2], Yue Cao [3 4 *]

[1] Department of Pediatric Dentistry, Hospital of Stomatology, Zhongshan City, No. 73 Hubin Road, Shiqi Street, Zhongshan City, Guangdong Province, 528400, P. R. China

[2] The Second School of Clinical Medicine, Southern Medical University, Guangzhou 510515, Guangdong, China

[3] Department of Cardiology, The Affiliated Hospital of Southwest Medical University, Luzhou, Sichuan, China

[4] The Second School of Clinical Medicine, Southern Medical University, Guangzhou 510515, Guangdong, China. caoyuegege@yeah.net

[*] **Corresponding author:** Yue Cao (clinical_scientist@yeah.net)

Qi Luo: qllq1114@163.com

Qian Yang: 21920514@smu.edu.cn

Yue Cao: clinical_scientist@yeah.net

Abstract

Background: Nutritional status, recognized as a modifiable determinant of oral health, has recently gained increasing attention in the context of edentulism. The triglyceride–total cholesterol–body weight index (TCBI) is a novel nutritional indicator derived from routine clinical measures. However, its association with edentulism remains unclear. This study was designed to assess the association between TCBI and edentulism risk.

Methods: This study utilized survey data provided by the China Health and Retirement Longitudinal Study (CHARLS). Three analyses were performed: cross-sectional (n = 9,686), prospective (participants without baseline edentulism and with incident edentulism, n = 8,568), and trajectory analyses (TCBI trajectories and incident edentulism, n = 4,921). Logistic regression, Cox proportional hazards models, group-based trajectory modeling, and restricted cubic spline analyses were applied. Sensitivity analyses using cumulative TCBI during follow-up were also conducted.

Results: In the cross-sectional analysis, individuals in the highest TCBI tertile demonstrated a significantly lower risk of prevalent edentulism (adjusted OR = 0.80, 95% CI: 0.65–0.97). In the prospective analysis, higher TCBI levels were independently associated with a reduced risk of incident edentulism (adjusted HR = 0.85, 95% CI: 0.77–0.92). Trajectory modeling demonstrated that individuals with persistently high TCBI had the lowest risk of incident edentulism (adjusted HR = 0.59,

95% CI: 0.40–0.89). These associations remained robust in sensitivity analyses.

Conclusion: TCBI was consistently and inversely associated with edentulism across cross-sectional, prospective, and trajectory analyses. As a readily obtainable nutritional index, TCBI may have clinical utility for the early identification and risk prediction of edentulism.

Keywords: Tooth Loss; Triglyceride–Cholesterol–Body Weight Index; Nutritional Status; Prospective Studies; Longitudinal Studies

Introduction

Edentulism, defined as the complete loss of all natural teeth, has been recognized as the ultimate marker of oral health burden (1). According to the World Health Organization, approximately 23% of individuals aged 60 years and above are completely edentulous. Edentulism not only compromises masticatory function and nutritional intake but also contributes to speech difficulties, social withdrawal, and psychological decline, imposing a substantial burden on older adults (2,3). Recent studies have indicated that tooth loss represents not merely a localized oral condition but also a significant indicator of systemic health risks. Evidence has shown strong associations between tooth loss and cardiovascular disease, frailty, cognitive decline, and increased all-cause mortality (4–6). These findings highlight that the prevention and management of tooth loss extend beyond dental care, becoming a crucial issue in public health and systemic disease prevention. Identifying modifiable risk factors for edentulism and promoting early screening and prevention are therefore essential for improving population health.

The development of edentulism is typically a cumulative process influenced by multiple factors, including dental caries, periodontal disease, poor oral hygiene, smoking, low socioeconomic status, and limited access to dental care (7,8). Beyond these well-established determinants, nutritional status—a modifiable lifestyle factor—has recently been recognized as a potential contributor to tooth preservation and oral tissue homeostasis (9). Adequate nutrition supports collagen synthesis, bone remodeling, and immune defense, maintaining the stability of periodontal and alveolar

structures. Conversely, malnutrition is associated with impaired collagen formation, reduced immune function, and accelerated alveolar bone resorption, which may hasten the transition from partial tooth loss to complete edentulism (10–12). Epidemiological studies have further shown that individuals with poorer baseline nutritional status are more likely to develop extensive tooth loss or edentulism, suggesting that declining nutrition may play a critical role in this progressive process (13,14). Thus, edentulism not only represents the terminal stage of dental loss but may also reflect the cumulative effects of long-term nutritional deficiency.

However, traditional single-nutrient or anthropometric indicators are insufficient to comprehensively assess an individual's overall nutritional reserves (15,16). The triglyceride–cholesterol–body weight index (TCBI), a composite nutritional indicator derived from routine clinical parameters, provides an objective reflection of energy and nutritional status (17). Previous research has demonstrated that low TCBI levels are closely associated with cardiovascular events, adverse cancer outcomes, and higher all-cause mortality, underscoring its value as an integrated marker of systemic nutrition (18–20). Nonetheless, no studies to date have investigated the relationship between TCBI and edentulism, and its potential implications for oral health remain unexplored.

Building on this background, the present study hypothesized that lower TCBI levels might be linked to an increased risk of edentulism. Using nationally representative data from the China Health and Retirement Longitudinal Study (CHARLS) (21), this analysis comprehensively assessed the association between TCBI and edentulism through cross-sectional, prospective, and trajectory analyses.

Considering that edentulism represents a long-term cumulative process, both static and dynamic aspects of nutritional status were examined. By integrating these complementary analytical designs, the present study sought to clarify the population-level association between overall nutritional status and the development of edentulism, provide new epidemiological evidence for the role of nutrition in maintaining oral health, and inform early screening and individualized prevention strategies.

Methods

Data source

Information for this study was sourced from CHARLS, a longitudinal cohort that collects data from Chinese adults aged 45 years and older and is designed to achieve national population coverage (21). The initial round of data collection took place in 2011–2012 and was based on a population-based sampling framework. Subsequent follow-up waves were carried out every two to three years to gather updated information on participants' demographic characteristics, socioeconomic conditions, health status, and lifestyle behaviors.

Study design and population

At baseline (wave 2011), 17,705 participants were enrolled. For the cross-sectional analysis, participants lacking data on edentulism (n = 139), TCBI (n = 7,766), or key covariates (n = 114) were excluded, leaving 9,686 participants. The prospective and trajectory analyses were subsequently conducted within this remaining population. For

the prospective analysis, participants with edentulism at baseline (n = 838) or without follow-up information (n = 280) were further excluded, yielding 8,568 participants. For the trajectory analysis, participants missing TCBI data in wave 2015, those who developed edentulism between wave 2011 and 2015, or those lacking wave 2018 follow-up data (n = 4,765) were excluded, resulting in 4,921 participants. A schematic overview of participant inclusion and exclusion is provided in Figure 1.

Exposure variable: TCBI

TCBI was derived according to the following equation (22):

$$\text{TCBI} = \frac{\left(\text{triglycerides (mg/dL)} \times \text{ total cholesterol (mg/dL)} \times \text{ body weight (kg)}\right)}{1000}$$

Outcome variable: Edentulism

Edentulism was assessed through the oral health questionnaire using the item, "Have you lost all of your natural teeth?" A response of "yes" to this question indicated edentulism, whereas a response of "no" indicated non-edentulism (23). Self-reported edentulism has been validated as a reliable measure in older populations and is widely adopted in large-scale epidemiological studies (24,25).

Covariates

Covariate selection was grounded in prior empirical evidence (21,26) and informed by a directed acyclic graph (DAG) constructed to illustrate and evaluate the hypothesized causal relationships among TCBI, edentulism, and potential confounders

(Supplementary Figure 1). The final model included age, sex, residence (rural/urban), education (primary school, middle school, college or above, or no formal education), marital status (unmarried, married, or others), smoking status (yes/no), drinking status (yes/no), hypertension (ascertained through respondents' prior medical diagnosis or elevated blood-pressure readings at the survey examination), and diabetes (identified on the basis of previously diagnosed disease or abnormal glucose measurements obtained during the survey). Multicollinearity was assessed using the generalized variance inflation factor (GVIF) (Supplementary Table 1-2). To allow comparisons across predictors with differing degrees of freedom, $GVIF^{(1/(2 \cdot Df))}$ values were examined, and all covariates showed values below 2, indicating no evidence of meaningful multicollinearity (27).

Statistical analysis

Baseline characteristics were compared according to edentulism status. Categorical variables were assessed using chi-square–based procedures. Continuous variables were analyzed according to their underlying data distributions. For group comparisons, Student's t test or the Wilcoxon rank-sum test was applied to evaluate differences between two independent groups, whereas one-way analysis of variance or the Kruskal–Wallis test was employed for comparisons involving three or more groups, as dictated by distributional assumptions.

For the cross-sectional analysis, multivariable logistic regression was applied to evaluate the association between TCBI and edentulism. Restricted cubic spline (RCS)

models were used to explore possible nonlinear associations between baseline TCBI and edentulism.

For the prospective cohort analysis, baseline TCBI (wave 2011) was used to evaluate its longitudinal association with incident edentulism during follow-up. Baseline TCBI (wave 2011) was categorized into tertiles (T1–T3) to ensure balanced subgroup sizes and facilitate comparison across nutritional strata. Multivariable Cox proportional hazards models quantified the hazard ratios (HRs) and corresponding 95% confidence intervals (CIs). RCS models were also applied to evaluate dose–response relationships in the longitudinal context.

For the trajectory analysis, repeated TCBI measurements from waves 2011 and 2015 were incorporated into group-based trajectory modeling (GBTM) to characterize long-term intraindividual changes and their associations with edentulism risk. Models with one to four trajectories were fitted, and a three-group solution (Low, Moderate, High) was selected after considering the lowest Bayesian Information Criterion (BIC), acceptable Average Posterior Probability of Assignment (APPA ≥ 0.70), minimum group size >5%, and clinical interpretability (28,29) (Supplementary Table 3 and Supplementary Figure 2). Multivariable Cox proportional hazards models were applied to determine whether higher TCBI levels corresponded to a reduced hazard of developing edentulism over time.

To verify the stability of the longitudinal results, a sensitivity analysis was carried out using cumulative TCBI as an alternative exposure metric. This analysis was performed within the same dataset used for the trajectory analysis, which includes two

TCBI measurements (2011 and 2015) and complete follow-up information for incident edentulism. Cumulative TCBI was calculated by averaging the two measurements, and Cox proportional hazards models were applied to re-evaluate the association with incident edentulism.

Statistical analyses adopted a two-sided testing strategy, with a P value threshold of 0.05 defining statistical significance. Analyses were carried out in R (version 4.4.0), with the relevant packages summarized in Supplementary Table 4.

Results

Cross-sectional Analysis: Participant Characteristics

In the cross-sectional analysis, 9,686 individuals were eligible for inclusion, of whom 8,848 did not have edentulism and 838 were identified with edentulism (Table 1). Those classified with edentulism showed markedly reduced TCBI values compared with individuals without edentulism (6.91 vs. 7.06, $P < 0.001$). The median age was substantially higher among participants with edentulism (69.00 vs. 58.00 years, $P < 0.001$). A greater proportion of individuals with edentulism resided in rural areas compared with those without edentulism (71.36% vs. 64.22%, $P < 0.001$). Regarding education, a markedly greater proportion of individuals with edentulism had no formal education (65.87% vs. 45.91%, $P < 0.001$). The proportion of married individuals was significantly lower among those with edentulism (74.46% vs. 89.46%, $P < 0.001$). For lifestyle factors, smoking prevalence was similar between groups (41.29% vs. 38.44%, $P = 0.11$), alcohol consumption was less frequent in participants with edentulism (28.16%

vs. 32.78%, $P = 0.007$). As for comorbidities, hypertension was more prevalent among those with edentulism (47.26% vs. 40.14%, $P < 0.001$), while diabetes was less common (3.70% vs. 5.96%, $P = 0.009$). No meaningful difference in sex distribution was detected between the two groups (P = 0.42).

Cross-sectional Analysis: Association Between TCBI and Edentulism

As shown in Table 2, when treated as a continuous measure, TCBI displayed a significant inverse association with edentulism in the unadjusted model (OR = 0.69, 95% CI: 0.62–0.77, $P < 0.001$). This inverse association persisted after stepwise adjustment for covariates (OR = 0.86, 95% CI: 0.76–0.96, $P = 0.009$). In the tertile analysis, individuals in the highest tertile (T3) exhibited a significantly lower risk of edentulism than those in the lowest tertile (T1) (OR = 0.80, 95% CI: 0.65–0.97, $P = 0.03$). A test for trend indicated a decreasing risk of edentulism with increasing TCBI levels (P for trend = 0.03). RCS modeling further confirmed this linear inverse association (P overall = 0.02), and no significant non-linear effect was observed (P non-linear = 0.37) (Figure 2).

Prospective Cohort Analysis: Baseline Characteristics

According to Table 3, 8,568 participants were included in the prospective cohort and stratified into tertiles based on their TCBI levels: T1 (n = 2,860), T2 (n = 2,852), and T3 (n = 2,856). Age distribution differed significantly across TCBI tertiles, with median ages of 58.00 years in T1, 58.00 years in T2, and 57.00 years in T3 ($P < 0.001$).

Sex distribution also varied, with the highest proportion of females in T3 (56.42%) and a relatively lower proportion in T1 (50.51%, $P < 0.001$). Educational attainment differed across TCBI tertiles. Participants without formal education were most common in T1 (49.32%) and became progressively fewer in T2 (45.65%) and T3 (42.07%) ($P < 0.001$). Residence also differed significantly across tertiles, with rural proportions decreasing from 70.17% (T1) to 65.15% (T2) and 59.00% (T3), while urban proportions increased accordingly ($P < 0.001$). Regarding marital status, the proportion of married participants was highest in T3 (91.74%) and lowest in T1 (88.08%, $P < 0.001$). For lifestyle characteristics, both smoking prevalence ($P < 0.001$) and drinking prevalence ($P = 0.04$) declined with increasing TCBI. In terms of clinical conditions, the prevalence of hypertension was highest in T3 (51.77%) and lowest in T1 (28.84%, $P < 0.001$). Similarly, diabetes prevalence increased across groups (3.39% vs. 5.26% vs. 8.93%, $P < 0.001$). The prevalence of edentulism was highest in T1 (16.22%) and lowest in T3 (11.76%, $P < 0.001$).

Prospective Cohort Analysis: Association Between TCBI and Incident Edentulism

In the prospective cohort, higher TCBI levels were significantly associated with a reduced risk of edentulism (Table 4). When analyzed as a continuous variable, the unadjusted model showed that each unit increase in TCBI was associated with a significantly lower risk of edentulism (HR = 0.77, 95% CI: 0.71–0.84, $P < 0.001$). This inverse association persisted after stepwise adjustment for demographic factors, lifestyle behaviors, and comorbidities (fully adjusted HR = 0.85, 95% CI: 0.77–0.92, P

< 0.001). In the tertile analysis, participants in the highest TCBI tertile (T3) demonstrated a significantly lower risk of edentulism relative to those in the lowest tertile (T1) (HR = 0.82, 95% CI: 0.71–0.95, P = 0.009). A test for trend indicated a significant decreasing risk of edentulism with increasing TCBI levels (P for trend = 0.009). RCS modeling further demonstrated an approximately linear relationship between TCBI and edentulism risk (P overall < 0.001), with no detectable evidence of non-linearity (P non-linear = 0.87) (Figure 3).

Trajectory Analysis: Baseline Characteristics

In the trajectory analysis cohort, 4,921 participants were included and subsequently classified into the low-TCBI group (n = 1,639), moderate-TCBI group (n = 2,461), and high-TCBI group (n = 821) (Figure 4, Table 5). With increasing TCBI levels, participants were younger and the proportion of females increased, with median ages of 58.00, 57.00, and 56.00 years in the low-, moderate-, and high-TCBI groups, respectively (P < 0.001). The proportion of females was highest in the high-TCBI group (59.56%, P < 0.001). Educational attainment also varied significantly, with a higher proportion of middle school or higher education in the high-TCBI group, whereas nearly half of participants in the low-TCBI group had no formal education (49.91%, P < 0.001). Residence also differed across groups, with the proportion of rural residents highest in the low-TCBI group (71.81%) and lowest in the high-TCBI group (56.64%) (P < 0.001). In addition, the proportion of married individuals was highest in the high-TCBI group (94.40%, P < 0.001), and the prevalence of smoking declined progressively

with increasing TCBI (34.59%, P < 0.001). Differences in drinking status were relatively small, with only a slightly higher prevalence in the low-TCBI group compared with the others (35.02%, P = 0.04). Regarding clinical characteristics, the prevalence of hypertension (53.35%, P < 0.001) and diabetes (10.48%, P < 0.001) was significantly higher in the high-TCBI group. Moreover, TCBI values measured in both 2011 and 2015 increased progressively across the groups (both P < 0.001), while the prevalence of edentulism decreased gradually with increasing TCBI (P < 0.001) (Table 5).

Trajectory Analysis: Association Between TCBI Trajectories and Edentulism Risk

In the trajectory analysis cohort, participants with higher TCBI levels had a significantly lower risk of edentulism (Table 6). Compared with the low-TCBI group, the moderate-TCBI group showed a lower risk in the unadjusted model (HR = 0.73, 95% CI: 0.57–0.92, P = 0.009); however, this association was attenuated and lost statistical significance after stepwise adjustment for demographic characteristics and lifestyle factors (fully adjusted HR = 0.80, 95% CI: 0.63–1.02, P = 0.07). In contrast, the high-TCBI group consistently demonstrated a protective effect across models, with an unadjusted HR of 0.48 (95% CI: 0.32–0.71, P < 0.001) and a fully adjusted HR of 0.59 (95% CI: 0.40–0.89, P = 0.01). These findings suggest that a robust reduction in edentulism risk was observed only at higher TCBI levels.

Sensitivity Analyses

In the sensitivity analyses, participants were categorized according to cumulative TCBI levels. The results showed that the incidence of edentulism decreased progressively with higher cumulative TCBI levels (Supplementary Table 5, $P < 0.001$). Multivariable regression further confirmed that higher cumulative TCBI levels were independently associated with a lower risk of edentulism (HR = 0.67, 95% CI: 0.50–0.91, $P = 0.01$). These results aligned with the primary analyses, suggesting that the conclusions were robust (Supplementary Table 6).

Discussion

Using data from a large, nationally representative cohort, this study provides the first comprehensive evaluation of TCBI in relation to edentulism across cross-sectional, prospective, and trajectory analyses. Higher TCBI levels were consistently associated with a lower risk of edentulism, and individuals maintaining higher TCBI over time exhibited the greatest protection. RCS analyses further indicated a monotonic decrease in the probability of edentulism with increasing TCBI levels. These findings suggest that TCBI, a simple and cost-effective nutritional index derived from routine clinical parameters, may serve as a practical tool for identifying individuals at high risk of edentulism and for guiding early nutrition-based preventive strategies in clinical and public health practice.

Existing reviews and population-based studies have consistently demonstrated a stable association between nutritional status and edentulism. Multiple systematic reviews in older adults have reported that individuals with partial or complete

edentulism exhibit a higher prevalence of malnutrition or nutritional risk. Although heterogeneity exists among studies in terms of assessment methods and adjustment for confounding variables, the overall direction of association remains consistent (9). Prospective cohort studies have also shown that individuals with reduced dentition often experience decreased dietary diversity and poorer diet quality, both of which are closely linked to insufficient intake of energy, protein, and micronutrients (30,31). These findings suggest a close interrelationship between nutritional status and changes in dentition. Broader epidemiologic evidence has further indicated that edentulism is strongly associated with malnutrition and a decline in overall health status (32), implying that long-term nutritional inadequacy may play a crucial role in the progressive loss of teeth leading to complete edentulism.

Building upon this foundation, the present study adds to existing evidence by applying a multidimensional analytical framework. The consistent inverse association observed across cross-sectional, longitudinal, and trajectory analyses strengthens the evidential credibility of the findings and helps mitigate the temporal constraints inherent to traditional cross-sectional designs. Notably, the trajectory analysis revealed that sustained declines in TCBI were associated with the highest risk of edentulism, offering new evidence for the cumulative impact of long-term nutritional insufficiency on oral health. Compared with earlier studies focusing on single nutrient indicators or self-reported dietary habits, TCBI—derived from objective clinical measurements—provides a more comprehensive and reproducible reflection of overall nutritional reserves.

Lifestyle behaviors—including dietary patterns, smoking, alcohol consumption, and physical activity— have been widely examined in relation to tooth loss; however, their explanatory power at the population level remains limited. These behaviors are subject to socioeconomic, cultural, and adherence-related variability, and inconsistencies in measurement methods have led to poor reproducibility (33–35). Recent systematic reviews have also highlighted considerable methodological heterogeneity and limited certainty of evidence regarding the relationship between health behaviors and tooth loss (35). In contrast, TCBI offers several methodological advantages, including objectivity, standardization, quantifiability, and accessibility. Multiple studies across diverse disease domains have validated its clinical relevance, showing that lower TCBI levels are strongly associated with higher risks of cardiovascular events, adverse cancer outcomes, and all-cause mortality (36–38). Collectively, these findings indicate that TCBI provides a more comprehensive reflection of overall nutritional reserves and demonstrates strong reproducibility and external applicability.

The clinical and public health significance of this study lies in the potential of TCBI, a composite nutritional index derived from routine clinical parameters, to serve as a simple, objective, and cost-effective tool for oral health risk assessment. Its integration into primary care and community screening programs could enable earlier recognition of people likely to be at elevated risk of edentulism and provide an evidence-based framework for nutritional intervention and personalized health management.

Strengths and Limitations

This study has several notable strengths. Leveraging CHARLS, a large and nationally representative cohort, increased the population-level relevance and generalizability of the findings. The multidimensional analytical framework, which incorporated cross-sectional, prospective, and trajectory analyses, provided a rigorous and comprehensive evaluation of both static and dynamic nutritional effects on edentulism. The use of TCBI as an objective and reproducible nutritional index derived from routine clinical measurements improved measurement accuracy compared with self-reported dietary data. In addition, advanced statistical techniques, including RCS and trajectory modeling, enabled precise assessment of dose–response relationships and long-term nutritional patterns.

Despite the large sample size and multidimensional design, certain study constraints remain. First, although multiple covariates were adjusted for, the possibility of residual confounding cannot be fully excluded. For instance, the CHARLS database does not include information on oral hygiene practices or oral health behaviors that may influence tooth loss, and these variables could play important roles in the development of edentulism. The absence of such data may have introduced unmeasured confounding, potentially affecting the stability and broader applicability of the results. Second, edentulism status was determined based on self-reported information rather than clinical examination, and detailed data on the number of remaining teeth or periodontal condition were unavailable. This limitation may have introduced minor measurement bias; nevertheless, prior research assessing the accuracy of self-reported oral conditions

has indicated that older adults' reports of tooth loss correspond well with outcomes obtained from clinical evaluations (24,25). Third, because the analysis was based on a Chinese cohort, the external validity of the findings should be interpreted with caution.

Additional investigations are needed to corroborate the present results in heterogeneous populations and incorporate clinical oral examinations, objective dental records, and oral hygiene behavior data. In addition, prospective interventional studies are warranted to determine whether improving nutritional status and increasing TCBI levels can reduce the risk of edentulism, thereby providing direct evidence to inform prevention and management strategies.

Conclusions

Higher TCBI levels were consistently associated with a lower risk of edentulism across cross-sectional, prospective, and trajectory analyses. As a simple and objective nutritional indicator, TCBI may help identify individuals with poor nutritional reserves who are at increased risk of tooth loss and may support earlier preventive dental care. Further studies, including interventional research and validation in diverse populations, are needed to determine whether improving nutritional status or modifying TCBI levels can slow or prevent the progression of edentulism.

Abbreviations

TCBI: triglyceride–cholesterol–body weight index

CHARLS: China Health and Retirement Longitudinal Study

RCS: restricted cubic spline

TG: triglyceride

OR: odds ratio

BMI: body mass index

TC: total cholesterol

GBTM: group-based trajectory modeling

CI: confidence interval

HR: hazard ratio

DAG: directed acyclic graph

GVIF: generalized variance inflation factor

Declarations

Ethics approval and consent to participate

This study was carried out using publicly accessible, de-identified data from the China Health and Retirement Longitudinal Study (CHARLS). Ethical review for the original CHARLS project was provided by the Biomedical Ethics Review Committee of Peking University (IRB00001052-11015), and informed consent was obtained from participants at enrollment. As the present investigation analyzed anonymized secondary datasets without any direct involvement of human subjects, additional institutional ethical approval was not required.

Competing interests

The authors declare no competing interests.

Consent for publication

No separate consent for publication was necessary.

Acknowledgements

The directed acyclic graph (DAG) illustrating the hypothesized causal relationships in this study was created using the online tool DAGitty (http://www.dagitty.net).

Funding

This study did not rely on any dedicated external financial support.

Availability of data and materials

This study relied on de-identified information provided by the China Health and Retirement Longitudinal Study (CHARLS). Access to the CHARLS dataset can be requested by eligible researchers through the official application portal (http://charls.pku.edu.cn) after completing the required registration procedures. Requests for research materials related to this work may be directed to the corresponding author, who will provide access when the inquiry is considered appropriate.

Authors' contributions

Qi Luo: Conceptualization, Methodology, Formal analysis, Writing – original draft; **Qian Yang**: Data curation, Investigation, Validation, Writing – original draft; **Yue Cao**: Supervision, Project administration, Resources, Writing – review & editing. All authors read and approved the final manuscript.

20251218013750119884367412281753

ORIGINALITY REPORT

14%
SIMILARITY INDEX

PRIMARY SOURCES

1	www.frontiersin.org<br>Internet	120 words — 3%
2	storage.googleapis.com<br>Internet	91 words — 2%
3	www.researchsquare.com<br>Internet	62 words — 1%
4	Dongli Huang, Hang Wu, Yanhua Huang. "Novel indicator for erectile dysfunction: the CALLY index, evidence from data of NHANES 2001-2004", Frontiers in Endocrinology, 2025<br>Crossref	47 words — 1%
5	pmc.ncbi.nlm.nih.gov<br>Internet	33 words — 1%
6	bmcpublichealth.biomedcentral.com<br>Internet	25 words — 1%
7	www.science.gov<br>Internet	24 words — 1%
8	Guimei Li, Shujuan Li. "Exploring the prognostic value of the novel nutritional index for in-hospital mortality in acute coronary syndrome: a sex-specific analysis", Frontiers in Medicine, 2025<br>Crossref	16 words — < 1%

9	pesquisa1.bvsalud.org<br>Internet	16 words — < 1%
10	Herden, Carla Carlotta. "Predicting Individuals' Intention to Make Socially Responsible Investments : Application of an Extended Theory of Planned Behavior", Universidade Catolica Portuguesa (Portugal), 2024<br>ProQuest	15 words — < 1%
11	i2b.us<br>Internet	14 words — < 1%
12	ideas.repec.org<br>Internet	12 words — < 1%
13	wjgnet.com<br>Internet	12 words — < 1%
14	www.mdedge9-ma1.mdedge.com<br>Internet	12 words — < 1%
15	www.wjgnet.com<br>Internet	12 words — < 1%
16	www.nature.com<br>Internet	11 words — < 1%
17	assets-eu.researchsquare.com<br>Internet	10 words — < 1%
18	behcetuzdergisi.com<br>Internet	10 words — < 1%
19	www.e-crt.org<br>Internet	10 words — < 1%

20 synapse.koreamed.org
Internet
9 words — < 1%

21 worldwidescience.org
Internet
9 words — < 1%

22 www.medrxiv.org
Internet
9 words — < 1%

23 Huang Chen Chang, Jun-Peng Chen, Yi-Ming Chen, Wen-Nan Huang Yi-Hsing Chen. "ANTI-C1Q ANTIBODIES AS INDICATORS OF DISEASE ACTIVITY, RENAL INVOLVEMENT, AND NON-SCARRING ALOPECIA IN PATIENTS WITH SLE", The Journal of Rheumatology, 2025
Crossref
8 words — < 1%

24 Persitz, Jonathan. "Impact of Time-to-Surgery on Adverse Outcomes for Distal Radius Fractures: A Population-Based Study.", University of Toronto (Canada)
ProQuest
8 words — < 1%

25 aura.abdn.ac.uk
Internet
8 words — < 1%

26 Huaqing Liu, Julie E Byles, Xiaoyue Xu, Min Zhang, Xuesen Wu, John J Hall. "Evaluation of successful aging among older people in China: Results from China health and retirement longitudinal study", Geriatrics & Gerontology International, 2017
Crossref
6 words — < 1%

27 Tang, Wang-Choi. "Sodium Glucose Co-Transporter 2 Inhibitor and the Risk of Ventricular Arrhythmia Among People With Type 2 Diabetes", McGill University (Canada), 2025
ProQuest
6 words — < 1%

28 Xu Liu, Jing Wang, Juxiang Jin, Yan Tong, Qiu Fu, Te Zhao, Shuai Wang. "Association between the C-reactive protein-triglyceride glucose index and incident hypertension across different blood pressure states: findings from the CHARLS", Springer Science and Business Media LLC, 2025
Crossref Posted Content

6 words — < 1%

EXCLUDE QUOTES	OFF	EXCLUDE SOURCES	OFF
EXCLUDE BIBLIOGRAPHY	ON	EXCLUDE MATCHES	OFF
