# Supplementary material for: A novel nutritional index and risk of edentulism: evidence from cross-sectional, prospective, and trajectory analyses
Source: Lipids Health Dis. 2026 Jan 14;25:50. doi: 10.1186/s12944-026-02860-2 (PMC12888110; doi:10.1186/s12944-026-02860-2)
Supplement: Supplementary file 5 — Supplementary Material 5. Supplementary Tables [file 12944_2026_2860_MOESM5_ESM.docx]

**Supplementary Table 1. Generalized variance inflation factor (GVIF) for multicollinearity assessment in the cross-sectional logistic regression model.**

| **Variable** | **Df** | **GVIF** | **GVIF^(1/(2*Df))** |
| --- | --- | --- | --- |
| TCBI | 1 | 1.089084 | 1.043592 |
| Age | 1 | 1.319048 | 1.148498 |
| Sex | 1 | 2.149709 | 1.466188 |
| Education | 3 | 1.264921 | 1.039945 |
| Marital status | 2 | 1.225028 | 1.052050 |
| Residence | 1 | 1.076087 | 1.037346 |
| Smoke | 1 | 1.827976 | 1.352027 |
| Drinking | 1 | 1.225027 | 1.106810 |
| Hypertension | 1 | 1.095881 | 1.046843 |
| Diabetes | 1 | 1.021775 | 1.010829 |

GVIF, generalized variance inflation factor; Df, degree of freedom; TCBI, triglyceride–total cholesterol–body weight index.

All variables had GVIF^(1/(2*Df)) < 2, indicating no significant multicollinearity.

**Supplementary Table 2.Generalized variance inflation factor (GVIF) for multicollinearity assessment in the prospective Cox regression model.**

| **Variable** | **Df** | **GVIF** | **GVIF^(1/(2*Df))** |
| --- | --- | --- | --- |
| TCBI | 1 | 1.099204 | 1.048429 |
| Age | 1 | 1.340632 | 1.157857 |
| Sex | 1 | 2.203566 | 1.484441 |
| Education | 3 | 1.277078 | 1.041605 |
| Marital status | 2 | 1.199588 | 1.046545 |
| Residence | 1 | 1.077371 | 1.037965 |
| Smoke | 1 | 1.871624 | 1.368073 |
| Drinking | 1 | 1.259244 | 1.122160 |
| Hypertension | 1 | 1.112262 | 1.054638 |
| Diabetes | 1 | 1.029255 | 1.014522 |

**Supplemental Table 3. Model fit statistics for group-based trajectory modeling of depressive Symptoms.**

| **Model**  **(No. of Groups)** | **AIC** | **BIC** | **Min Group Size (%)** | **Max Group Size (%)** | **APPA** |
| --- | --- | --- | --- | --- | --- |
| 1 | 21080.83 | 21102.41 | 100 | 100 | 1 |
| 2 | 19072.65 | 19123.01 | 44.46 | 55.54 | 0.8924 / 0.8944 |
| 3 | 18429.54 | 18501.49 | 19.14 | 47.30 | 0.8041 / 0.8602 / 0.8516 |
| 4 | 18230.02 | 18330.74 | 10.39 | 38.86 | 0.7827 / 0.7826 / 0.7863 / 0.8385 |

**Supplementary Table 4.Main R packages used in statistical analysis.**

| **Package** | **Version** | **Purpose** |
| --- | --- | --- |
| stats | 4.4.0 | Logistic regression and basic statistical analysis |
| survival | 3.5-8 | Cox proportional hazards model analysis |
| gbmt | 0.1.4 | Group-based trajectory modeling (GBTM) |
| compareGroups | 4.8.0 | Baseline characteristics and group comparisons |
| gtsummary | 1.7.2 | Regression results summarization and presentation |
| car | 3.1-2 | Variance Inflation Factor (VIF) for collinearity assessment |
| rcssci | 0.4.0 | Visualization of restricted cubic splines (RCS) |

**Supplementary Table 5. Baseline characteristics in the cumulative TCBI analysis.**

|  | **T1(15.76, 20.56)**  **n = 1641** | **T2(20.56, 22.26)**  **n = 1640** | **T3(22.26, 29.52)**  **n = 1640** | ***P* vaule** |
| --- | --- | --- | --- | --- |
| **Age** | 58.00 [51.00;64.00] | 57.00 [50.00;63.00] | 56.00 [50.00;61.00] | <0.001 |
| **Sex:** |  |  |  | <0.001 |
| **Female** | 826 (50.34%) | 963 (58.72%) | 974 (59.39%) |  |
| **Male** | 815 (49.66%) | 677 (41.28%) | 666 (40.61%) |  |
| **Education:** |  |  |  | <0.001 |
| College or above | 17 (1.04%) | 22 (1.34%) | 23 (1.40%) |  |
| Middle school | 436 (26.57%) | 524 (31.95%) | 545 (33.23%) |  |
| No formal education | 818 (49.85%) | 701 (42.74%) | 682 (41.59%) |  |
| Primary school | 370 (22.55%) | 393 (23.96%) | 390 (23.78%) |  |
| **Residence:** |  |  |  | <0.001 |
| Rural | 1179 (71.85%) | 1073 (65.43%) | 992 (60.49%) |  |
| Urban | 462 (28.15%) | 567 (34.57%) | 648 (39.51%) |  |
| **Marital status:** |  |  |  | <0.001 |
| Married | 1469 (89.52%) | 1509 (92.01%) | 1519 (92.62%) |  |
| Others | 155 (9.45%) | 128 (7.80%) | 118 (7.20%) |  |
| Unmarried | 17 (1.04%) | 3 (0.18%) | 3 (0.18%) |  |
| **Smoke:** |  |  |  | <0.001 |
| No | 976 (59.48%) | 1073 (65.43%) | 1082 (65.98%) |  |
| Yes | 665 (40.52%) | 567 (34.57%) | 558 (34.02%) |  |
| **Drinking:** |  |  |  | 0.01 |
| No | 1059 (64.53%) | 1118 (68.17%) | 1135 (69.21%) |  |
| Yes | 582 (35.47%) | 522 (31.83%) | 505 (30.79%) |  |
| **Hypertension:** |  |  |  | <0.001 |
| No | 1186 (72.27%) | 1021 (62.26%) | 813 (49.57%) |  |
| Yes | 455 (27.73%) | 619 (37.74%) | 827 (50.43%) |  |
| **Diabetes:** |  |  |  | <0.001 |
| No | 1584 (96.53%) | 1557 (94.94%) | 1506 (91.83%) |  |
| Yes | 57 (3.47%) | 83 (5.06%) | 134 (8.17%) |  |
| **TCBI wave 2011** | 6.48 [6.25;6.71] | 7.09 [6.86;7.31] | 7.80 [7.51;8.15] | <0.001 |
| **TCBI wave 2015** | 6.55 [6.31;6.77] | 7.17 [6.95;7.40] | 7.86 [7.56;8.22] | <0.001 |
| **Cumulative TCBI** | 19.66 [19.06;20.13] | 21.38 [20.97;21.80] | 23.37 [22.76;24.29] | <0.001 |
| **Edentulism:** |  |  |  | 0.001 |
| No | 1518 (92.50%) | 1538 (93.78%) | 1567 (95.55%) |  |
| Yes | 123 (7.50%) | 102 (6.22%) | 73 (4.45%) |  |

**Supplementary Table 6. Association between cumulative TCBI and edentulism.**

| **Characteristic** | **Model 1**  **HR (95% CI) ^*^ *P* value** | **Model 2**  **HR (95% CI) ^*^ *P* value** | **Model 3**  **HR (95% CI) ^*^ *P* value** |
| --- | --- | --- | --- |
| **TCBI**  **(Continuous)** | 0.88 (0.83, 0.94)  <0.001 | 0.92 (0.86, 0.98)  0.009 | 0.91 (0.85, 0.97)  0.005 |
| **TCBI quartile** |  |  |  |
| T1 | Ref | Ref | Ref |
| T2 | 0.82 (0.63, 1.07)  0.15 | 0.91 (0.70, 1.19)  0.51 | 0.90 (0.69, 1.18)  0.45 |
| T3 | 0.58 (0.44, 0.78)  <0.001 | 0.69 (0.52, 0.93)  0.02 | 0.67 (0.50, 0.91)  0.01 |
| **P for trend** | <0.001 | 0.02 | 0.01 |
| ^1^HR = Hazard Ratio, CI = Confidence Interval  Model 1: Non-adjusted.  Model 2: Adjusted for age, sex, education, marital status, smoke, drinking, residence.  Model 3: Further adjusted for hypertension and diabetes based on model 2. | | | |
